# Epidemiology of Mucinous Adenocarcinomas

**DOI:** 10.3390/cancers12113193

**Published:** 2020-10-30

**Authors:** Matthew G. K. Benesch, Alexander Mathieson

**Affiliations:** Discipline of Surgery, Faculty of Medicine, Memorial University of Newfoundland, St. John’s, NL AlB 3V6, Canada; alex.mathieson@med.mun.ca

**Keywords:** colloid carcinoma, histopathology, chemotherapy, radiotherapy, surgery, cancer

## Abstract

**Simple Summary:**

Mucinous adenocarcinomas are a very uncommon type of cancer that is poorly studied. These cancers overexpress a jelly-like substance called mucin outside their cells. These cancers share characteristics to another group of cancers called signet ring cell adenocarcinomas, which express mucin inside their cells, and these cancers almost invariability have a worse outcome compared to conventional adenocarcinomas. In our study, we show that patient outcomes with mucinous adenocarcinomas depend largely on the site of the cancer, with both similarities and differences to cancers arising from signet ring cells. This work, along with our recent study on signet ring cell cancers, provides a solid epidemiological reference for future work to motivate investigations as to how recognizing this tumor histology should lead to more tailored cancer treatments in order to improve patient outcomes.

**Abstract:**

Mucinous (colloid) adenocarcinomas (MAs) are a rare histological subtype of adenocarcinomas where extracellular mucin comprises more than 50% of the tumor. Most literature on MAs relate to cancers from colorectal and breast sites; however, the literature lacks a standardized overview of the MA disease entity. Particularly in colorectal cancer, some MAs may have signet ring cells floating within the mucin, which may represent a highly metastatic phenotype. MAs and signet ring cell adenocarcinomas represent a spectrum of mucin-producing neoplastic conditions where in the latter most mucin is intracellular rather than extracellular. We recently published a standardized overview of signet ring cells, and in this companion work, using a retrospective cohort approach, we summarize the clinicodemographic and mortality outcomes of MAs in sixteen primary sites, comprising 95.6% of all MAs in the Surveillance, Epidemiology, and End Results Program (SEER), a population-level cancer database covering nearly one-third of the United States population. Compared to matching nonvariant adenocarcinomas, MAs have a slightly earlier age of onset with increased rates of regional and distant disease at presentation. Survival outcomes are highly dependent on tumor location, illustrating our poor understanding of MA tumor biology. The clinical significance of MA histology depends largely on tumor site.

## 1. Introduction—Overview of Mucinous Adenocarcinomas

**Figure 1:**
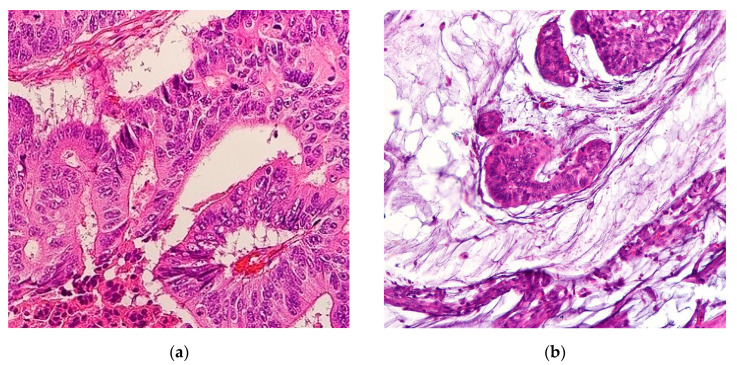
(**a**) Representative histological slide of colorectal adenocarcinoma. (**b**) Representative histological slide of colorectal mucinous adenocarcinoma, illustrating abundant extracellular mucin within more than 50% of the tumor area. Figures sourced from Wikimedia Commons, public domain [8,9].

Mucinous adenocarcinomas (MAs), also referred to as colloid carcinomas, are cancers where >50% of the tumor is comprised of extracellular mucin with overt malignant epithelial cells in clumps, layers, or individual cells [1] (Figure 1). Carcinomas with mucinous areas under 50% are categorized as having a mucinous component [1]. Mucin is a high-molecular-weight glycoprotein that has oligosaccharides attached to a core protein. Physiologically, mucin is produced by epithelial mucosal cells to lubricate and provide a protective barrier against outside pathogens and noxious substances [2]. Mucin also stores biologically active molecules in reserve for inflammatory and wound-healing functions [3]. Deregulation of mucin core protein expression in some adenocarcinomas leads to aberrant forms and amounts of mucin expression, enabling cancer cells to subvert their cell signaling physiological roles into pro-growth and survival defense states against the immune system [2,4]. Abundant mucin disrupts cell–cell interactions thereby promoting cell plasticity and anchorage-independent growth, conditions both necessary for invasion and metastasis [5]. A similar process occurs in the cousin histological subtype of MA, the signet ring cell adenocarcinoma (SRCC). In SRCCs, an even rarer cancer than MAs, mucin is instead produced intracellularly in greater than 50% of cancer cells, displacing the nucleus to periphery, therefore giving the cell the overall appearance of a signet ring [6]. In gastrointestinal tract malignancies, signet ring cells are a common component with MAs. The location of the mucin majority, whether intracellularly or extracellularly, primarily determines the histological subtype of the adenocarcinomas [1]. Hence MAs and SRCCs are considered on opposite ends of the mucin neoplasm spectrum [2]. These morphologies are categorized by the International Classification of Disease for Oncology (ICD)-0–3 classification as 8480/3 for MA and 8490/3 for SRCC under the umbrella of Cystic, Mucinous and Serous Neoplasms [7]. 

Like SRCCs, MAs are extremely rare, collectively comprising about 1% of cancer diagnoses. Outside of colorectal and breast cancers, the two most common sites, our knowledge of MAs depends largely on small case series, and there is no study that systematically compares MAs to matching nonvariant cases. We recently published a summary of the clinicodemographic and mortality outcomes of SRCCs using the Surveillance, Epidemiology, and End Results (SEER) database [6]. In this study, we employ the same database and study variables for MAs. This will allow readers and investigators to directly compare and contrast MAs and SRCCs to each other and nonvariant cases.

The SEER database is a population-based cancer registry managed by the National Cancer Institute, encompassing about one-third of the United States with near universal capture of cases as regional registries were added to the program since 1975 [10]. It is an instrumental resource for comparing histopathological data with survival and mortality data across cancer sites, demographics, and time [11]. In this retrospective site-stratified analysis, using the SEER database, we investigate the clinicopathological characteristics and survival outcomes of MAs and compare them to all other cancers by site, with subgroup analysis to major nonvariant types. 

## 2. Results—Analysis of Mucinous Adenocarcinomas by Site

**Figure 2:**
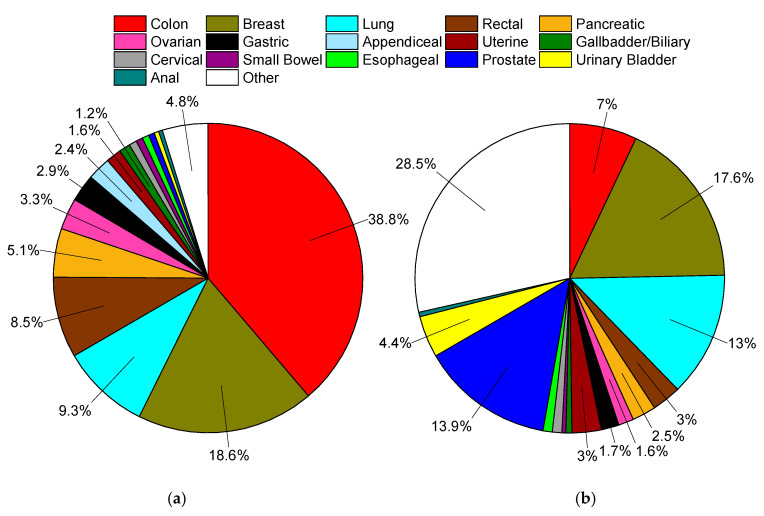
(**a**) Distribution of mucinous adenocarcinomas in Surveillance, Epidemiology, and End Results Program (SEER), 1975–2016, total of 169,595 cases. (**b**) Distribution of all solid (non-blood borne), non-mucinous tumors in SEER, 1975–2016, total of 9.44 million cases. In both plots data labels are percentages. Markers omitted if less than 1%.

Subsections are presented in order of the percentage of MA cases relative to all cases of MA (Figure 2), and gynecological cancers in Tables S1 and S2. First presented within each subsection is a demographics table with all included cases of cancers for that site, followed by the most common nonvariant histological type(s), and then MA cases. Incidence rates and age-adjusted cause-specific and relative survivals are also presented. The second table presents both univariate and multivariable analyses for cause-specific mortality according the same demographic, histopathological, and treatment variables as the first table. Here, we present the hazard ratios for MAs compared to all other non-MAs within the site of interest. We then provide a sub-analysis of nonvariant histological type(s) to MAs to provide a representative comparator of risk to common cancer types within each site.

**Figure 3:**
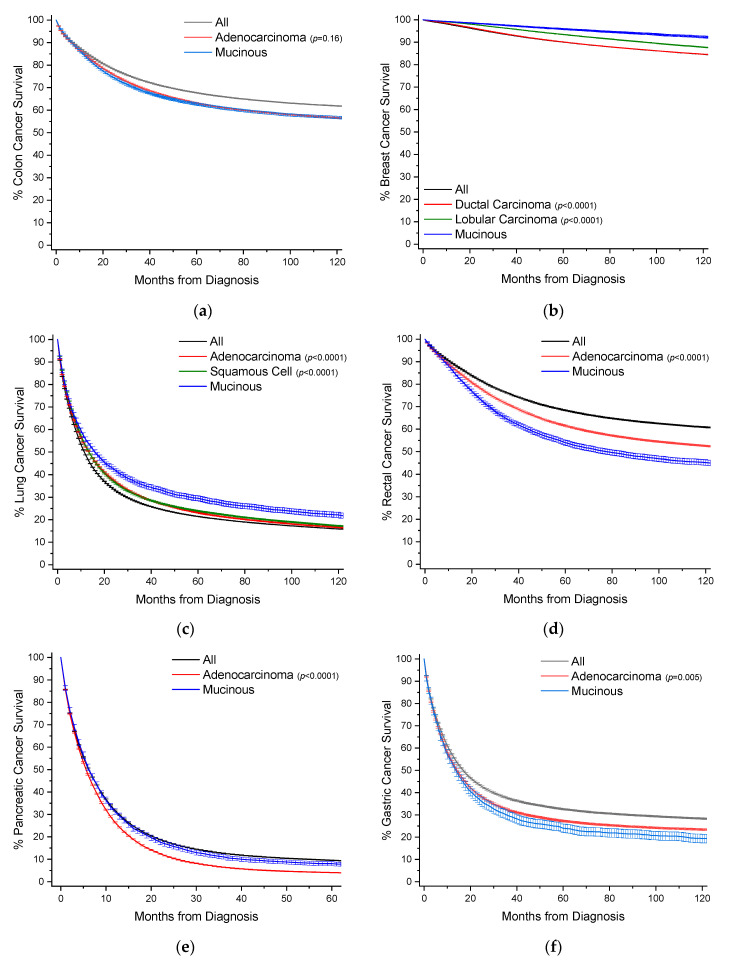

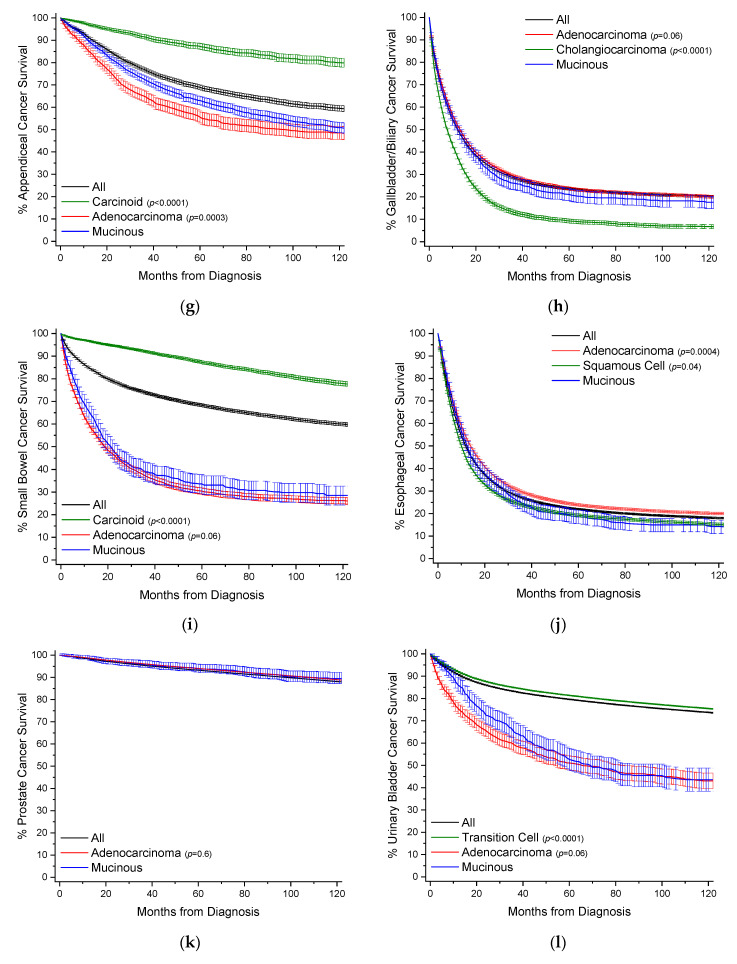

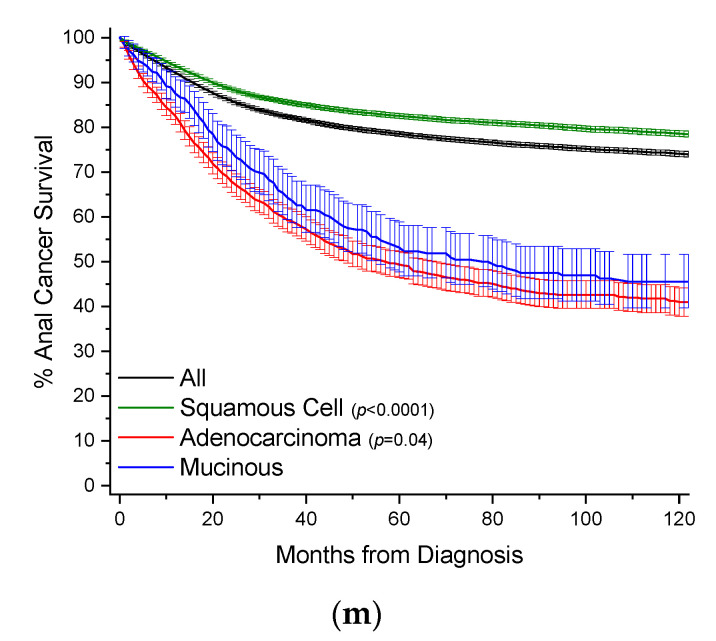
Kaplan–Meier survival curves. All survivor functions are shown with 95% confidence intervals. (**a**) Colon cancer. (**b**) Breast cancer. (**c**) Lung cancer. (**d**) Rectal cancer. (**e**) Pancreatic cancer. (**f**) Gastric cancer. (**g**) Appendiceal cancer. (**h**) Gallbladder/Biliary cancer. (**i**) Small Bowel cancer. (**j**) Prostate cancer. (**k**) Prostate cancer. (**l**) Urinary Bladder cancer. (**m**) Anal cancer. In these curves, “All” represents the curves for all cancers within that site, with subtypes shown as labelled. Among subtypes, pairwise statistical comparisons by the log-rank test is shown relative to mucinous adenocarcinomas.

To provide a visual overview across all cancer sites, Kaplan–Meier survival curves with 95% confidence intervals are also presented, with *p*-values in comparison to MAs (Figure 3). 

### 2.1. Colon

Colon cancers are analyzed separately from cancers arising from the appendix and rectum (Section 2.4 and Section 2.7). Carcinoids rather than nonvariant adenocarcinomas are the most common cancer subtype in the appendix, and rectal cancer treatment often requires radiotherapy, unlike colon cancers. MAs comprise 9.0% of all cases of colon cancer, and nearly 39% of all MA cases (Table 1, Figure 2). When compared to nonvariant adenocarcinomas, MAs have virtually the same age of onset. The proportion of female cases increases from 51.1% to 53.9%. These cancers are most often detected at a regional stage (46.1% vs. 41.5%). Survival time is statistically the same at both 5 and 10 years (Figure 3a, Table 1). When compared to all other colon cancers, MAs have a hazard ratio (HR) of mortality of 1.06 (95% CI: 1.04–1.08) and 1.04 (95% CI: 1.02–1.06) to adenocarcinomas, after multivariable analyses (Table 2).

### 2.2. Breast

MAs comprise 1.8% of all cases of breast cancer, and nearly 19% of all MA cases (Table 3, Figure 2). When compared to ductal and lobular cancers, MAs have an older age of onset by nearly 7 years, unique among the MAs. Most MAs present as localized disease (84.2% vs. ~40–50% for ductal and lobular cancers), and are well differentiated (46.0% vs. ~16–18%). Owing to less aggressive disease at presentation, MAs have better 5-year (96% vs. ~90%) and 10-year (92% vs. ~85%) survivals relative to both ductal and lobular cancers (Figure 3b, Table 3). This is also reflected in multivariable analyses, where the HR of mortality for MAs compared to both ductal and lobular cancers is about 0.70 (95% CI: 0.66–0.75) (Table 4).

### 2.3. Lung

MAs comprise 1.2% of all cases of lung cancer, and 9.3% of all MA cases (Table 5, Figure 2). MAs have an earlier age of onset of 0.9 years and 2.9 years relative to nonvariant adenocarcinomas and squamous cells, respectively. Compared to adenocarcinomas, MAs have roughly the same gender distribution, and are less likely to present with distant disease (49.5% vs. 58.4%). Like breast cancers, lung MAs are more likely to present with well and moderate grades than lung adenocarcinomas (~47% vs. ~25%). Consequently, MA patients are more likely to receive surgery (46.5% vs. 28.3%). Median survival time is about 15.8 months, compared to about 12.5 months for both squamous cells and adenocarcinomas (Figure 3c, Table 5). However, after multivarable analysis, MAs have a HR of mortality of 1.19 (95% CI: 1.16–1.22) compared to all other cancers, 1.23 (95% CI: 1.20–1.26) to adenocarcinomas, and 1.07 (95% CI: 1.04–1.10) to squamous cells (Table 6).

### 2.4. Rectal

MAs comprise 4.6% of all cases of rectal cancers, and 8.5% of all MA cases (Table 7, Figure 2). When compared to nonvariant adenocarcinomas, MAs have a slightly earlier mean age of onset of 0.6 years. There is no difference in gender distribution. MAs are more likely to be detected as regional disease (52.6% vs. 42.6%) with poor differentiation (18.3% vs. 13.2%). Patients with MAs are more likely to receive surgery (88.3% vs. 79.3%), radiotherapy (52.1% vs. 46.3%), and chemotherapy (61.7% vs. 56.6%) than patients with nonvariant adenocarcinomas. Rectal MA patients have worse overall survival at 5 years (53.1% vs. 59.8%) and 10 years (44.1% vs. 50.7%), respectively (Figure 3d, Table 7), with a median survival time of 72.2 months. When compared to all rectal cancers, MAs have a HR of mortality of 1.35 (95% CI: 1.31–1.39), and 1.28 (95% CI: 1.24–1.32) to nonvariant adenocarcinomas, after multivariable analyses (Table 8).

### 2.5. Pancreatic

MAs comprise 3.5% of all cases of pancreatic cancer, and 5.1% of all MA cases (Table 9, Figure 2). Pancreatic MA patients have a mean age of about one year younger than nonvariant adenocarcinomas (66.9 vs. 67.8 years). There is also a slight gender bias towards females (51.6% vs. 48.7%). MAs have higher rate of distant disease at detection compared to adenocarcinomas (65.1% vs. 58.6%); however they have higher rates of surgical resection (24.3% vs. 16.7%). Median survival is only marginally better (6.3 vs. 5.6 months), with better 5-year (8.0% vs. 3.8%) and 10-year (6.1% vs. 2.5%) survival rates (Table 9, Figure 3e). When compared to all other pancreatic cancers, MAs have the same HR for mortality, and slightly better compared to nonvariant adenocarcinomas at 0.87 (95% CI: 0.84–0.90), after multivariable analyses (Table 10).

### 2.6. Gastric

MAs comprise 2.4% of all cases of gastric cancer, and nearly 2.9% of all MA cases (Table 11, Figure 2). When compared to nonvariant adenocarcinomas, MAs have an earlier age of onset by about 1 year. Gender proportion is the same between both groups. These cancers are more likely to be detected at a regional/distant stage (75.5% vs. 66.4%). These patients are more likely to receive surgery (61.1% vs. 51.2%), though there are no differences in use of radiotherapy or chemotherapy. Survival is slightly worse compared to adenocarcinomas at 5 years (23.3% vs. 25.8%) and 10 years (18.7% vs. 22.7%) (Figure 3f, Table 11). Median survival time is 13.0 months for MAs and 13.5 months for adenocarcinomas. When compared to all other gastric cancers, MAs have a HR of mortality of 1.16 (95% CI: 1.10–1.22), and 1.09 (95% CI: 1.04–1.15) to adenocarcinomas, after multivariable analyses (Table 12).

### 2.7. Appendiceal

MAs comprise 28.5% of all cases of appendiceal cancer, and 2.4% of all MA cases (Table 13, Figure 2). Comparative data for carcinoid tumors is presented as they are the most common cancer subtype at 39.8% of cases. When compared to nonvariant adenocarcinomas, MAs have an earlier mean age of onset 3.4 years. There is a female gender bias compared to adenocarcinomas (55.1% vs. 47.1%). MAs are much more likely to present with distant stage disease relative to adenocarcinomas (53.9% vs. 29.9%), but are more likely to present as well differentiated (33.5% vs. 13.7%) rather than poorly differentiated 8.8% vs. 21.0%). There are no real differences in application of treatment modalities. By 10 years, survival rates are equal between both groups at ~47.5% with a median survival time between 90–100 months (Figure 3g, Table 13). After multivariable analyses, relative to all other appendiceal cancers, MAs have a HR of mortality of 0.81 (95% CI: 0.74–0.87), 1.23 (95% CI: 1.08–1.40) to carcinoids, and 0.64 (95% CI: 0.57–0.71) to nonvariant adenocarcinomas (Table 14).

### 2.8. Gallbladder/Biliary

MAs comprise 3.0% of all cases of gallbladder/biliary cancer, and 1.2% of all MA cases (Table 15, Figure 2). Compared to the most two common subtypes, nonvariant adenocarcinoma and cholangiocarcioma, MAs have an earlier age of onset of 1–1.5 years. There is no gender bias between MAs and nonvariant adenocarcinomas. MAs have the highest rate of distant stage at detection at 38.8%, compared to 31.5% and 36.8% for nonvariant adenocarcinomas and cholangiocarcinomas, respectively. MAs have higher rates of surgery relative to adenocarcinomas (67.5% vs. 60.8%). Survival times between the two groups are essentially the same, with median survival at 1 year (Figure 3h, Table 15). Similarly, when compared to all other gallbladder/biliary cancers, MAs have a HR of mortality of 1.08 (95% CI: 1.01–1.16), and non-significant to nonvariant adenocarcinomas and cholangiocarcinomas (Table 16).

### 2.9. Small Bowel

MAs comprise 2.6% of all cases of small bowel cancer, and 0.71% of all MA cases (Table 17, Figure 2). Data for carcinoid tumors is presented as this is the most common cancer subtype at 53.4% of cases. MAs have an earlier mean age of onset of 1.9 years compared to nonvariant adenocarcinomas, and 2 years later than carcinoid tumors. There is no overt gender bias. There are no great differences in detection stage between MAs and adenocarcinomas, but MAs do present with slightly better stages overall. MA patients have much higher rates of surgery (75.7% vs. 60.1%) relative to adenocarcinomas. MAs have better 5-year (32.6% vs. 27.4%) and 10-year (27.6% vs. 23.1%) survival rates compared to nonvariant adenocarcinomas (Figure 3i, Table 17). Median survival time is 20.3 months for MAs and 16.2 months for adenocarcinomas. When compared to all small bowel cancers, MAs have a HR of mortality of 1.67 (95% CI: 1.51–1.86), 4.39 (95% CI: 3.79–5.09) to carcinoids, and statistically the same to nonvariant adenocarcinomas, after multivariable analyses (Table 18).

### 2.10. Esophageal

MAs comprise 1.4% of all cases of esophageal cancer, and 0.70% of all cases of MA (Table 19, Figure 2). When compared to nonvariant adenocarcinomas, MAs have a slightly earlier age of onset by about 10 months. There is no overt gender bias. Similarly, there are no larger differences in detection stage or grade, however MAs are more likely to present with regional disease (38.0%) but poor grade whereas nonvariant adenocarcinomas present mostly with distant disease (37.4%). MA patients are more likely to receive surgery, radiotherapy, and chemotherapy than adenocarcinomas patients. MA have worse 5-year (17.3% vs. 21.9% vs. 18.3%) and 10-year (14.2% vs. 17.8% vs. 14.6%) survival rates than either adenocarcinomas or squamous cells, respectively (Figure 3j, Table 19). Median survival time is 12.3 months for MAs, compared to 9.7 months and 13.2 months for squamous cells and adenocarcinomas, respectively. MAs have a HR of mortality of 1.15 (95% CI: 1.06–1.24) compared to all other esophageal cancers, 1.19 (95% CI: 1.10–1.29) to adenocarcinomas, and statistically the same to squamous cells, after multivariate analyses (Table 20).

### 2.11. Prostate

MAs comprise 0.1% of all cases of prostate cancer, and 0.62% of all cases of MA (Table 21, Figure 2). When compared to nonvariant adenocarcinomas, which comprise 96.0% of all cases of prostate cancer, MAs have an earlier mean age of onset of 2.8 years. While the stage of disease at detection is roughly the same between the two groups, MAs are more likely to present with poor grade (48.1% vs. 37.3%). MA patients receive surgery in 68.5% of cases, compared to 44.4% of nonvariant adenocarcinomas. Instead, adenocarcinoma patients are more likely to receive radiotherapy (35.1% vs. 24.4%). There is no difference in survival rates between the two groups (Figure 3k, Table 21). When compared to nonvariant adenocarcinomas, there is no difference in HR of mortality for MAs (Table 22).

### 2.12. Urinary Bladder

MAs comprise 0.2% of all cases of urinary bladder cancer, and 0.49% of all MA cases (Table 23, Figure 2). Transition cell carcinomas comprise 94% of all urinary bladder cases, and nonvariant adenocarcinomas 0.5% of all cases. Mean age of onset is younger for MAs by 10.2 years and 7.2 years compared to transition cell carcinomas and adenocarcinomas, respectively. MAs primarily present with localized and regional disease; however, predominantly with a well grade differentiation. MA patients receive more surgery than adenocarcinoma patients (94.4% vs. 85.5%). MAs have comparable 5-year survival rates to adenocarcinomas at ~50% (Figure 3l, Table 23). Median survival time is 62.9 months for MAs, and 57.6 months for adenocarcinomas. When compared to all other urinary cancers, MAs have a HR of mortality of 2.39 (95% CI: 2.10–2.71), on univariate analysis, but non-significantly 1.13 (95% CI: 0.99–1.28) after multivariate analyses (Table 24).

### 2.13. Anal

MAs comprise 1.3% of all cases of anal cancer, and 0.38% of all MA cases (Table 25, Figure 2). Squamous cell carcinomas comprise 75.5% of all cases of anal cancer and are included for comparison purposes. Mean age of onset for MA cases is about 1 year earlier compared to adenocarcinomas, and nearly 11 years later than squamous cell cancers. MAs have a large male gender bias compared to the other subtypes (61.3% vs. ~51%). MAs most often present as regional disease in 48.4% of all cases, compared to 34.4% for nonvariant adenocarcinomas, with a bias towards poor grade (20.3% vs. 16.4%). Similar to most other MA types, anal MA patients receive more surgery compared to nonvariant adenocarcinomas (78.7% vs. 60.3%). Survival rates between MAs and adenocarcinomas are relatively similar (Figure 3m, Table 25). When compared to all other anal cancers, MAs have a HR of mortality of 2.38 (95% CI: 2.04–2.77), 1.41 (95% CI: 1.21–1.67) compared to squamous cell cancers, and statistically the same compared to nonvariant adenocarcinomas (Table 26).

### 2.14. Gynecological Cancers (Ovarian, Uterine, Cervical)

Gynecological cancers comprise about 5.9% of all MAs, and MAs comprise 3.7%, 1.0%, and 1.6% of all cases of ovarian, uterine, and cervical cancers, respectively (Table S1, Figure 2). Given both their rarity among MAs and numerous histological subtypes, we provide comparisons of only MAs to all other cancers within their respective anatomical sites, to facilitate broad comparisons of the presentation and outcomes of MAs in these sites to other locations. When compared to all ovarian cancers, ovarian MAs present 4.6 years earlier (56.1 years vs. 60.7 years), whereas ages of presentation are similar among uterine and cervical cancers (Table S1). Across all three sites, MAs are more likely to present with localized disease, and better grades than other cancer histotypes. MAs have better 5-year survival rates for ovarian (51.8% vs. 40.6%) and uterine cancers (87.1% vs. 78.0%), but worse for cervical cancer (51.8% vs. 57.5%) (Table S1). After multivariable analyses, the HR of mortality for MAs is 1.52 (95% CI: 1.44–1.61) compared to all other ovarian cancers, equivalent in uterine cancers at 0.90 (95% CI: 0.79–1.03), and 1.49 (95% CI: 1.33–1.66) compared to all other cervical cancers (Table S2).

## 3. Discussion

This study represents the first extensive comparative analysis of MAs to site-matching nonvariant adenocarcinomas, and is a companion comparison to mucin-containing SRCCs [6]. MAs represent about 1% of all adenocarcinomas and occur at about four times the rate of SRCCs. Despite both cancer subtypes being defined by their mucin production, most MAs are colon cancers representing nearly 1/3 of all cases, whereas for SRCCs, gastric cancers represent over half of all cases [6]. Overall MAs have equivalent or slightly better survival rates compared to nonvariant adenocarcinomas, except for rectal cancers, which have worse outcomes after about 12 months. Rectal cancers, even after multivariate analyses, have a HR of mortality of about 1.3 relative to adenocarcinomas. Similarly, rectal SRCC patients had the worst relative outcomes with a HR of morality of 2.1 [6]. SRCCs have a large bias towards females with an earlier age of onset [6]. In comparison, this female bias is less for MAs compared to SRCCs, seen primarily in colon, pancreatic, and appendiceal cancers. Whereas SRCCs universally most often present with distant staged disease with poor grade, MAs present with a more mixed picture, with regional staged disease and moderate grade differentiation as the most common types. As a result, MA patients are more likely to be treated surgically that nonvariant adenocarcinoma or SRCC patients. It is worth noting that mucinous-like morphological changes are sometimes seen in tumors following neoadjuvant treatment; however, these should not be misdiagnosed as MAs [12].

Most of our understanding of MA biology comes from the two most prevalent sites involved, colon and breast. Consistent with our findings, a prognostic difference has not been established between MAs and nonvariant adenocarcinomas in colon cancer [13]. A population-based study of over 120,000 colon cancer patients in Europe showed that MA histology has no negative survival impact [14]. Even after accounting for tumor location, population characteristics, and treatment plans, the prognostic significance of MAs is colon cancer is uncertain [15]. However, in the cases of stage III and IV disease, MAs have a worse response to systemic treatment, likely related to the higher prevalence of microsatellite instability relevant to nonvariant adenocarcinomas [16,17]. Most colon MAs develop along the serrated pathway and are characterized by *BRAF* mutations and epigenomic instability [18]. These cancers have a tendency towards the right colon, which classically does not present symptoms until late in the disease state relative to left-sided cancers [19]. Further, some colon MA tumors contain signet ring cells floating within the mucin or attached to the adjacent stromal wall [20]. Unfortunately, this characteristic is not captured within SEER and is not typically reflected in the labeling of tumor histotype on pathology reports. Colorectal SRCCs tumors, like MAs, share higher rates of microsatellite instability, CpG island methylator phenotype-high (CIMP-H), and more frequent *BRAF* mutations [21]. In this same work, the authors demonstrated that even a small signet-ring cell component served as a poor prognostic indicator in these cancers, independent of other demographic and pathological features [21]. Therefore, the presence of any signet ring cells in MAs might serve to warrant specialized treatment plans for these patients. In agreement with the literature, we found that MAs of the rectum, unlike the colon, have worse outcomes compared to nonvariant adenocarcinomas event after adjustment for common clinicopathological parameters [22], but fortunately, this gap is narrowing since the introduction of preoperative short-term radiotherapy and chemotherapy in addition to total mesorectal excision surgery [15].

Unlike colon cancers, MA in breast cancer is a favorable outcome relative to most other varieties of breast cancer. The improved survival outcomes for breast MAs relative to more common ductal and lobular cancers may in part come from MAs having a lower rate of genetic instability [23]. Breast MAs exist in two large patterns, pure and mixed. Pure MAs are comprised of at least 90% mucin and are the most frequent pattern [24]. These tumors are more likely to carry the luminal A subtype and are rarely HER-2 positive, meaning that treatment by surgery and post-operative hormonal therapy is highly curative [25]. Despite both types typically presenting as localized disease, the mixed pattern is about 2.5 times more likely than the pure subtype to spread to lymph nodes [26]. Therefore, their management often requires an axillary dissection [27]. In breast cancers, MAs may initially be mistaken for benign lesions as a round shaped mass in mammography or a subcutaneous fat-like isoechoic appearance on ultrasound [28]. Consequently, it is important to keep this cancer entity in the differential when investigating apparently benign lesions.

This study does have several limitations. As a retrospective study, it is prone to selection bias. Treatment is encoded as a binary variable and does not account for temporal changes in advancements in therapeutics over the course of the quarter-century span covered within this study. This study has also had to use very broad definitions of tumor grade and stage to make analogous comparisons across all tumor sites. Because MAs are especially rare outside of colon and breast cancer, a population-level registry is necessary in order make a meaningful quantitative analysis. Consequently, the primary strength of this resource is our systematic analysis of one of the largest MA patient cohorts in a well-recognized and validated population level cancer registry that is highly regarded for its quality improvement methodology [11].

## 4. Materials and Methods

### 4.1. Patient Selection

The National Cancer Institute’s SEER database was employed and included all amalgamated data from all 18 SEER cancer registries from 1975–2016, covering 28% of the United States population. To keep our comparator populations consistent to those used in our previous publication on SRCCs, we have limited demographic and subsequent mortality analysis to diagnoses after 1992 [6]. Data release from the SEER database does not require informed patient consent. Permission to obtain the SEER database was obtained with the ID number 10095-Nov2018 via signed agreements [29].

A complete outline of exclusion criteria and its effect on case numbers is presented in Table S3. A summary of the 18 SEER cancer registries and count of cancer cases within each registry is shown in Table S4. A complete definition of all variables, sites analyzed, and an overview of rare non-analyzed sites are presented in Tables S5 and S6.

### 4.2. Statistical Analysis

All data from the 18 SEER cancer registries was imported into Stata 15.1 (StataCorp LLC, College Station, TX, USA) for statistical analysis from SEER 1975–2016 Research Data in ASCII text format. A complete case analysis was completed after variable definition in Table S3. Baseline patient characteristics were compared with the *t* or *χ^2^* test as appropriate. Univariate and multivariable Cox proportional hazard regression was used to determine the association of mortality with cancer histology type, after adjusting for age, gender, race, detection stage, grade differentiation, surgery, radiotherapy, and chemotherapy. All hazard ratios are calculated with 95% confidence intervals. Use of surgery, radiotherapy, and chemotherapy as treatment modalities are taken as binary variables. All *p*-values are two-sided, and the threshold of 0.05 was used to determine statistical significance. Survival curves were plotted using the Kaplan–Meier method. *p*-values for survival curves are generated using the log rank test. Graphs are produced using Origin Pro 2020 (OriginLab Corporation, Northampton, MA, USA). Incidence rates are calculated with SEER*Stat 8.3.6 (Surveillance Research Program, National Cancer Institute, Calverton, MD, USA), using SEER 18 (2000–2016 data) and are age-adjusted to the 2000 United Sates standard population with the age variable recode <1-year-olds. Cause-specific survival and relative survival are both age standardized to the International Cancer Survival Standard 1—Age 15+ variable via the actuarial method (and Ederer II cumulative expected method for relative survival), using SEER*Stat 8.3.6 and SEER 18 (2000–2016 data).

## 5. Conclusions

This study aims to provide a systematic and standardized characterization of the overall demographical and histopathological features of MAs across all major sites, offering comparison to nonvariant histological types. We have used the same study design as in our publication on SRCCs [6], thereby enabling researchers and clinicians to also directly compare MAs to SRCCs, which can have utility in future studies to improve understanding of rare cancers defined by mucin-production disorders. Future histopatholgical studies, particularly in colorectal cancers, need to account for the presence of any signet ring cell morphology, and ultimately identify tailored treatment modalities.

## Figures and Tables

**Table 1:** Baseline demographics and clinical characteristics by histology for colon cancers.

Colon	All	Adenocarcinoma	Mucinous
***N* (%)**	448,221 (100)	283,919 (63.3)	40,232 (9.0)
**Age (Years) (%)**			
0–14	32 (<0.1)	6 (<0.1)	5 (<0.1)
15–29	1930 (0.4)	1066 (0.4)	284 (0.7)
30–49	38,386 (8.6)	24,725 (8.7)	3829 (9.5)
50–69	182,891 (40.8)	113,023 (39.8)	14,582 (36.2)
70–85	172,601 (38.5)	111,469 (39.3)	16,609 (41.3)
>85	52,381 (11.7)	33,630 (11.8)	4923 (12.2)
**Mean (SD)**	68.4 (13.6)	68.7 (13.6)	68.9 (14.1)
**Gender (%)**			
Male	220,451 (49.2)	138,746 (48.9)	18,533 (46.1)
Female	227,770 (50.8)	145,173 (51.1)	21,699 (53.9)
**Race (%)**			
White	356,033 (79.4)	224,658 (79.1)	33,088 (82.2)
Black	55,559 (12.4)	34,885 (12.3)	4615 (11.5)
Other	36,629 (8.2)	24,376 (8.6)	2529 (6.3)
**Detection Stage (%)**			
In Situ	21,655 (4.8)	1975 (0.7)	12 (<0.1)
Localized	159,954 (35.7)	87,453 (30.8)	11,836 (29.4)
Regional	155,275 (34.6)	117,946 (41.5)	18,541 (46.1)
Distant	93,274 (20.8)	67,485 (23.8)	9392 (23.3)
Unstaged	18,063 (4.0)	9060 (3.2)	451 (1.1)
**Grade Differentiation (%)**			
Well	38,192 (8.5)	18,437 (6.5)	3765 (9.4)
Moderate	254,481 (56.8)	186,654 (65.7)	23,775 (59.1)
Poor	72,229 (16.1)	52,503 (18.5)	7564 (18.8)
Undifferentiated	7970 (1.8)	4397 (1.5)	759 (1.9)
Unknown	75,349 (16.8)	21,928 (7.7)	4369 (10.9)
**Surgery (%)**			
Yes	394,923 (88.1)	251,754 (88.7)	37,879 (94.2)
No	53,298 (11.9)	32,165 (11.3)	2353 (5.8)
**Radiotherapy (%)**			
Yes	9237 (2.1)	6971 (2.5)	1077 (2.7)
No	438,984 (97.9)	276,948 (97.5)	39,155 (97.3)
**Chemotherapy (%)**			
Yes	125,723 (28.0)	95,441 (33.6)	14,064 (35.0)
No	322,498 (72.0)	188,478 (66.4)	26,168 (65.0)
**Incidence Rate (95% CI)**			
All	31.2 (31.1–31.3)	20.1 (20.0–20.2)	2.85 (2.82–2.88)
Male	35.0 (34.9–35.2)	22.6 (22.5–22.7)	3.00 (2.96–3.05)
Female	28.1 (28.0–28.3)	18.15 (18.06–18.24)	2.70 (2.67–2.74)
**CSS % (95% CI)**			
1-year	83.5 (83.4–83.7)	83.3 (83.2–83.5)	84.1 (83.6–84.5)
2-year	75.9 (75.7–76.0)	74.8 (74.6–74.9) *	74.4 (73.9–74.9) *
5-year	64.6 (64.4–64.8)	61.8 (61.6–62.0) *	61.9 (61.3–62.5) *
10-year	58.5 (58.3–58.7)	55.0 (54.7–55.2) *	55.7 (55.0–56.4) *
Median (Months)	-	-	-
**RS % (95% CI)**			
1-year	82.1 (82.0–82.2)	81.9 (81.8–82.1)	83.0 (82.6–83.5)
2-year	74.8 (74.7–75.0)	73.8 (73.6–74.0) *	73.8 (73.2–74.3) *
5-year	64.2 (64.0–64.4)	61.7 (61.4–61.9) *	61.9 (61.2–62.6) *
10-year	57.8 (57.5–58.1)	54.7 (54.3–55.0) *	55.3 (54.3–56.2) *
Median (Months)	-	-	-

*p* < 0.05 for all comparisons between adenocarcinoma and mucinous adenocarcinoma, unless noted by * *p* ≥ 0.05. Incidence rates expressed per 100,000. SD, standard deviation; CSS, cause-specific survival; RS, relative survival; CI, confidence interval.

**Table 2:** Derived univariate and multivariable Cox-proportional hazard ratios (HR) of mortality for colon cancers.

Colon	Mucinous vs. Non-Mucinous		Mucinous vs. Adenocarcinoma	
HR (95% CI)	Univariate	Multivariable	Univariate	Multivariable
**Mucinous Histology**	1.20 (1.18–1.22)	1.06 (1.04–1.08)	1.01 (1.00–1.03) *	1.04 (1.02–1.06)
**Age (per 10 years)**	1.149 (1.145–1.154)	1.241 (1.235–1.246)	1.088 (1.083–1.093)	1.212 (1.206–1.218)
**Gender (Female)**	1.03 (1.02–1.05)	0.96 (0.95–0.97)	0.97 (0.96–0.99)	0.96 (0.95–0.97)
**Race**				
Black	1.22 (1.20–1.24)	1.21 (1.19–1.23)	1.29 (1.27–1.31)	1.23 (1.20–1.25)
Other	0.91 (0.89–0.92)	0.94 (0.92–0.95)	0.93 (0.91–0.95)	0.94 (0.92–0.96)
**Detection Stage**				
In Situ	0.42 (0.39–0.44)	0.39 (0.36–0.41)	0.53 (0.45–0.63)	0.44 (0.38–0.52)
Regional	3.01 (2.96–3.07)	3.12 (3.07–3.18)	2.48 (2.43–2.53)	2.68 (2.63–2.74)
Distant	17.5 (17.2–17.8)	16.6 (16.3–16.9)	14.2 (13.9–14.5)	14.4 (14.1–14.7)
Unstaged	8.75 (8.52–8.99)	4.32 (4.19–4.45)	6.69 (6.45–6.93)	3.45 (3.33–3.59)
**Grade Differentiation**				
Moderate	1.73 (1.68–1.77)	1.25 (1.22–1.28)	1.36 (1.32–1.39)	1.17 (1.14–1.21)
Poor	3.18 (3.10–3.26)	1.83 (1.79–1.88)	2.25 (2.19–2.32)	1.66 (1.61–1.71)
Undifferentiated	3.19 (3.05–3.33)	2.02 (1.94–2.11)	2.26 (2.15–2.38)	1.73 (1.64–1.82)
Unknown	2.28 (2.22–2.35)	1.40 (1.36–1.44)	3.59 (3.47–3.70)	1.38 (1.34–1.43)
**Surgery (Yes)**	0.167 (0.165–0.170)	0.388 (0.382–0.395)	0.173 (0.171–0.176)	0.398 (0.390–0.406)
**Radiotherapy (Yes)**	2.14 (2.08–2.20)	1.08 (1.05–1.11)	1.82 (1.77–1.88)	1.06 (1.03–1.10)
**Chemotherapy (Yes)**	1.95 (1.93–1.98)	0.86 (0.85–0.87)	1.58 (1.56–1.60)	0.83 (0.82–0.84)

*p* < 0.05 relative to reference unless noted by * *p* ≥ 0.05. Reference categories: Gender (Male), Race (White), Detection Stage (Localized), Grade differentiation (Well), Surgery (No), Radiotherapy (No), and Chemotherapy (No). CI, confidence interval.

**Table 3:** Baseline demographics and clinical characteristics by histology for breast cancers.

Breast	All	Ductal	Lobular	Mucinous
***N* (%)**	1,185,521 (100)	813,140 (68.6)	245,539 (20.7)	21,238 (1.8)
**Age (Years) (%)**				
0–14	17 (<0.1)	6 (<0.1)	0 (0)	0 (0)
15–29	6339 (0.5)	4862 (0.6)	508 (0.2)	88 (0.4)
30–49	283,566 (23.9)	202,015 (25.0)	55,048 (22.4)	3096 (14.6)
50–69	581,741 (49.1)	399,863 (49.0)	124,886 (50.9)	7945 (37.4)
70–85	265,451 (22.4)	176,716 (21.8)	55,945 (22.8)	8160 (38.4)
>85	48,407 (4.1)	29,678 (3.7)	9152 (3.7)	1949 (9.2)
**Mean (SD)**	60.2 (13.7)	59.7 (13.7)	60.7 (13.1)	66.7 (14.4)
**Gender (%)**				
Male	7059 (0.6)	5958 (0.7)	541 (0.2)	90 (0.4)
Female	1,178,012 (99.4)	807,182 (99.3)	244,998 (99.8)	21,148 (99.6)
**Race (%)**				
White	956,313 (80.7)	650,557 (80.0)	205,120 (83.5)	16,818 (79.2)
Black	124,772 (10.5)	88,018 (10.8)	21,734 (8.9)	2160 (10.2)
Other	104,436 (8.8)	74,565 (9.2)	18,685 (7.6)	2260 (10.6)
**Detection Stage (%)**				
In Situ	231,711 (18.0)	107,886 (13.3)	75,431 (30.7)	31 (0.1)
Localized	589,407 (49.7)	435,830 (53.6)	100,852 (41.1)	17,886 (84.2)
Regional	301,018 (25.4)	225,149 (27.7)	58,965 (24.0)	2566 (12.1)
Distant	63,128 (5.3)	36,705 (4.5)	8696 (3.5)	465 (2.2)
Unstaged	18,257 (1.5)	7570 (0.9)	1595 (0.6)	290 (1.4)
**Grade Differentiation (%)**				
Well	205,026 (17.3)	133,481 (16.4)	44,918 (18.3)	9775 (46.0)
Moderate	436,567 (36.8)	305,331 (37.5)	102,616 (41.8)	5965 (28.1)
Poor	357,949 (30.2)	287,496 (35.4)	42,394 (17.3)	876 (4.1)
Undifferentiated	28,401 (2.4)	18,932 (2.3)	5421 (2.2)	67 (0.3)
Unknown	157,578 (13.3)	67,900 (8.4)	50,190 (20.4)	4555 (21.4)
**Surgery (%)**				
Yes	1,105,455 (93.2)	768,601 (94.5)	233,092 (94.9)	20,168 (95.0)
No	80,066 (6.8)	44,539 (5.5)	12,447 (5.1)	1070 (5.0)
**Radiotherapy (%)**				
Yes	555,742 (46.9)	399,316 (49.1)	105,369 (42.9)	9845 (46.4)
No	629,779 (53.1)	413,824 (50.9)	140,170 (57.1)	11,393 (53.6)
**Chemotherapy (%)**				
Yes	387,251 (32.7)	297,100 (36.5)	61,058 (24.9)	2882 (13.6)
No	798,270 (67.3)	516,040 (63.5)	184,481 (75.1)	18,356 (86.4)
**Incidence Rate (95% CI)**				
All	68.1 (68.0–68.2)	48.6 (48.5–48.7)	12.69 (12.63–12.75)	1.52 (1.50–1.54)
Male	11.6 (11.3–11.9) **†**	9.4 (9.2–9.7) **†**	6.8 (6.1–7.4) **††**	1.3 (1.0–1.6) **††**
Female	126.3 (126.0–126.5)	90.4 (90.2–90.6)	23.5 (23.4–23.6)	2.74 (2.70–2.78)
**CSS % (95% CI)**				
1-year	96.35 (96.30–96.40)	97.21 (97.16–97.26)	97.96 (97.88–98.04)	99.0 (98.9–99.2)
2-year	93.57 (93.51–93.64)	94.5 (94.4–94.6)	96.1 (96.0–96.2)	98.3 (98.1–98.5)
5-year	87.0 (86.9–87.1)	88.0 (87.9–88.1)	90.1 (89.9–90.3)	95.9 (95.6–96.3)
10-year	80.5 (80.3–80.6)	81.7 (81.5–81.9)	82.6 (82.3–82.8)	92.4 (91.9–92.9)
Median (Months)	-	-	-	-
**RS % (95% CI)**				
1-year	96.93 (96.87–96.99)	97.93 (97.86–98.00)	98.9 (98.8–99.0)	99.6 (99.4–99.7)
2-year	94.7 (94.6–94.8)	95.8 (95.7–95.9)	97.8 (97.7–98.0)	99.4 (99.2–99.6)
5-year	89.2 (89.0–89.3)	90.3 (90.2–90.5)	93.3 (93.0–93.6)	98.6 (91.2–99.0)
10-year	83.0 (82.8–83.3)	84.5 (84.1–84.8)	86.4 (85.8–87.0)	95.9 (94.3–97.1)
Median (Months)	-	-	-	-

*p* < 0.05 for all comparisons among ductal, lobular, and mucinous adenocarcinoma. Incidence rates expressed per 100,000, except **†** (per 1 million), **††** (per 10 million). SD, standard deviation; CSS, cause-specific survival; RS, relative survival; CI, confidence interval.

**Table 4:** Derived univariate and multivariable Cox-proportional hazard ratios (HR) of mortality for breast cancers.

Breast	Mucinous vs. Non-Mucinous		Mucinous vs. Ductal		Mucinous vs. Lobular	
HR (95% CI)	Univariate	Multivariable	Univariate	Multivariable	Univariate	Multivariable
**Mucinous Histology**	0.49 (0.47–0.52)	0.68 (0.64–0.72)	0.50 (0.48–0.53)	0.71 (0.67–0.75)	0.63 (0.60–0.67)	0.70 (0.66–0.74)
**Age (per 10 years)**	1.149 (1.145–1.154)	1.219 (1.214–1.224)	1.09 (1.08–1.10)	1.198 (1.192–1.204)	1.24 (1.23–1.26)	1.28 (1.27–1.29)
**Gender (Male)**	1.61 (1.52–1.70)	1.08 (1.02–1.14)	1.61 (1.51–1.71)	1.11 (1.04–1.18)	1.21 (0.94–1.57) *	0.87 (0.68–1.13) *
**Race**						
Black	1.76 (1.73–1.78)	1.46 (1.44–1.48)	1.84 (1.81–1.87)	1.49 (1.47–1.52)	1.50 (1.45–1.57)	1.50 (1.44–1.56)
Other	0.80 (0.79–0.82)	0.88 (0.86–0.90)	0.84 (0.82–0.86)	0.87 (0.85–0.89)	0.69 (0.65–0.73)	0.88 (0.83–0.93)
**Detection Stage**						
In Situ	0.255 (0.246–0.263)	0.225 (0.217–0.233)	0.30 (0.29–0.31)	0.26 (0.25–0.27)	0.23 (0.21–0.24)	0.21 (0.20–0.23)
Regional	3.43 (3.39–3.47)	3.07 (3.03–3.11)	3.35 (3.30–3.40)	2.97 (2.93–3.02)	3.88 (3.77–4.00)	3.53 (3.41–3.65)
Distant	22.3 (22.0–22.6)	12.4 (12.2–12.6)	21.3 (20.9–21.7)	12.1 (11.8–12.3)	29.1 (28.0–30.2)	16.4 (15.7–17.1)
Unstaged	8.62 (8.40–8.85)	3.89 (3.78–4.00)	6.41 (6.15–6.68)	3.21 (3.08–3.36)	7.56 (6.91–8.26)	3.62 (3.29–3.97)
**Grade Differentiation**						
Moderate	2.31 (2.26–2.36)	1.81 (1.77–1.85)	2.52 (2.45–2.59)	1.94 (1.88–1.99)	1.67 (1.61–1.74)	1.41 (1.35–1.47)
Poor	4.68 (4.58–4.78)	3.17 (3.10–3.24)	5.08 (4.94–5.22)	3.44 (3.34–3.54)	2.66 (2.54–2.78)	2.23 (2.13–2.33)
Undifferentiated	2.48 (2.38–2.57)	3.35 (3.22–3.48)	2.62 (2.50–2.75)	3.52 (3.35–3.69)	0.98 (0.88–1.09) *	2.13 (1.91–2.37)
Unknown	3.70 (3.62–3.79)	2.46 (2.39–2.52)	2.82 (2.73–2.92)	2.50 (2.42–2.59)	1.61 (1.54–1.68)	1.63 (1.56–1.71)
**Surgery (Yes)**	0.118 (0.116–0.119)	0.393 (0.386–0.399)	0.128 (0.126–0.131)	0.39 (0.38–0.40)	0.134 (0.129–0.138)	0.37 (0.35–0.39)
**Radiotherapy (Yes)**	0.696 (0.689–0.703)	0.805 (0.796–0.814)	0.69 (0.68–0.70)	0.79 (0.78–0.80)	0.85 (0.83–0.88)	0.84 (0.82–0.86)
**Chemotherapy (Yes)**	2.22 (2.20–2.24)	1.09 (1.08–1.10)	2.15 (2.12–2.18)	1.06 (1.05–1.08)	2.64 (2.57–2.70)	1.21 (1.18–1.25)

*p* < 0.05 relative to reference unless noted by * *p* ≥ 0.05. Reference categories: Gender (Female), Race (White), Detection Stage (Localized), Grade differentiation (Well), Surgery (No), Radiotherapy (No), and Chemotherapy (No). CI, confidence interval.

**Table 5:** Baseline demographics and clinical characteristics by histology for lung cancers.

Lung	All	Adenocarcinoma	Squamous Cell	Mucinous
***N* (%)**	771,002 (100)	236,024 (30.6)	150,426 (19.5)	9325 (1.2)
**Age (Years) (%)**				
0–14	134 (<0.1)	3 (<0.1)	2 (<0.1)	2 (<0.1)
15–29	1125 (0.1)	191 (0.1)	67 (<0.1)	31 (0.3)
30–49	41,482 (5.4)	15,342 (6.5)	4915 (3.3)	755 (8.1)
50–69	356,243 (46.2)	116,978 (49.6)	68,258 (45.4)	4564 (48.9)
70–85	319,649 (41.5)	90,680 (38.4)	69,731 (46.4)	3555 (38.1)
>85	52,369 (6.8)	12,830 (5.4)	7453 (5.0)	418 (4.5)
**Mean (SD)**	68.3 (11.4)	67.2 (11.4)	69.2 (10.0)	66.3 (11.8)
**Gender (%)**				
Male	416,219 (54.0)	118,856 (50.4)	97,114 (64.6)	4676 (50.1)
Female	354,783 (46.0)	117,168 (49.6)	53,312 (35.4)	4649 (49.9)
**Race (%)**				
White	632,947 (82.1)	187,596 (79.5)	123,513 (82.1)	7722 (82.8)
Black	87,586 (11.4)	27,778 (11.8)	19,123 (12.7)	969 (10.4)
Other	50,469 (6.5)	20,650 (8.7)	7790 (5.2)	634 (6.8)
**Detection Stage (%)**				
In Situ	781 (0.1)	287 (0.1)	321 (0.2)	20 (0.2)
Localized	130,847 (17.0)	40,883 (17.3)	33,020 (22.0)	2432 (26.1)
Regional	185,572 (24.1)	50,483 (21.4)	53,745 (35.7)	2038 (21.9)
Distant	409,261 (53.1)	137,816 (58.4)	57,379 (38.1)	4614 (49.5)
Unstaged	44,541 (5.8)	6555 (2.8)	5961 (4.0)	221 (2.4)
**Grade Differentiation (%)**				
Well	31,217 (4.0)	13,206 (5.6)	3664 (2.4)	2019 (21.7)
Moderate	102,756 (13.3)	44,436 (18.8)	40,323 (26.8)	2423 (26.0)
Poor	187,802 (24.4)	68,573 (29.1)	53,169 (35.3)	1312 (14.1)
Undifferentiated	48,057 (6.2)	2123 (0.9)	1539 (1.0)	55 (0.6)
Unknown	401,170 (52.0)	107,686 (45.6)	51,731 (34.4)	3516 (37.7)
**Surgery (%)**				
Yes	189,192 (24.5)	66,710 (28.3)	46,764 (31.1)	4334 (46.5)
No	581,810 (75.5)	169,314 (71.7)	103,662 (68.9)	4991 (53.5)
**Radiotherapy (%)**				
Yes	297,248 (38.6)	92,699 (39.3)	70,553 (46.9)	2989 (32.1)
No	473,754 (61.4)	143,325 (60.7)	79,873 (53.1)	6336 (67.9)
**Chemotherapy (%)**				
Yes	311,134 (40.4)	101,041 (42.8)	56,174 (37.3)	3544 (38.0)
No	459,868 (59.6)	134,983 (57.2)	94,252 (62.7)	5781 (62.0)
**Incidence Rate (95% CI)**				
All	60.0 (59.9–60.1)	18.4 (18.3–18.4)	11.7 (11.6–11.7)	6.9 (6.8–7.1) **†**
Male	72.7 (72.4–72.9)	20.6 (20.5–20.7)	16.8 (16.7–16.9)	7.6 (7.4–7.9) **†**
Female	50.6 (50.4–50.8)	16.8 (16.7–16.9)	7.74 (7.67–7.80)	6.5 (6.3–6.7) **†**
**CSS % (95% CI)**				
1-year	46.6 (46.5–46.7)	50.9 (50.7–51.2)	51.2 (50.9–51.6)	55.5 (54.3–56.7)
2-year	31.8 (31.7–32.0)	36.0 (35.8–36.3)	35.2 (34.8–35.5)	42.6 (41.4–43.8)
5-year	20.2 (20.0–20.3)	21.6 (21.4–21.8)	22.5 (22.2–22.9)	28.8 (27.5–30.1)
10-year	14.9 (14.8–15.0)	15.1 (14.9–15.4)	16.2 (15.9–16.5)	21.4 (20.1–22.9)
Median (Months)	10.5	12.5	12.6	15.8
**RS % (95% CI)**				
1-year	44.5 (44.3–44.6)	49.5 (49.2–49.7)	48.8 (48.5–49.2)	54.2 (53.1–55.5)
2-year	29.8 (29.7–29.9)	34.6 (34.4–34.9)	32.6 (32.3–32.9)	41.5 (40.2–42.8)
5-year	18.1 (17.9–18.2)	20.1 (19.9–20.3)	19.5 (19.2–19.8)	27.9 (26.6–29.2)
10-year	12.1 (11.9–12.2)	13.0 (12.8–13.3)	11.6 (11.3–12.0)	19.8 (18.2–21.5)
Median (Months)	9.6	11.7	11.5	15.0

*p* < 0.05 for all comparisons among adenocarcinoma, squamous cell carcinoma, and mucinous adenocarcinoma. Incidence rates expressed per 100,000, except **†** (per 1 million). SD, standard deviation; CSS, cause-specific survival; RS, relative survival; CI, confidence interval.

**Table 6:** Derived univariate and multivariable Cox-proportional hazard ratios (HR) of mortality for lung cancers.

Lung	Mucinous vs. Non-Mucinous		Mucinous vs. Adenocarcinoma		Mucinous vs. Squamous Cell	
HR (95% CI)	Univariate	Multivariable	Univariate	Multivariable	Univariate	Multivariable
**Mucinous Histology**	0.81 (0.79–0.83)	1.19 (1.16–1.23)	0.87 (0.85–0.89)	1.23 (1.20–1.26)	0.88 (0.86–0.91)	1.07 (1.04–1.10)
**Age (per 10 years)**	1.114 (1.111–1.117)	1.112 (1.109–1.114)	1.068 (1.064–1.073)	1.094 (1.089–1.099)	1.087 (1.081–1.094)	1.073 (1.066–1.080)
**Gender (Female)**	0.827 (0.822–0.832)	0.842 (0.838–0.847)	0.80 (0.79–0.81)	0.83 (0.82–0.84)	0.88 (0.86–0.89)	0.88 (0.87–0.89)
**Race**						
Black	1.09 (1.08–1.10)	1.00 (0.99–1.01) *	1.09 (1.07–1.11)	1.00 (0.98–1.02) *	1.17 (1.15–1.19)	1.01 (0.99–1.03) *
Other	0.94 (0.93–0.95)	0.83 (0.82–0.84)	0.92 (0.90–0.94)	0.77 (0.75–0.78)	1.09 (1.06–1.12)	0.96 (0.94–0.99) *
**Detection Stage**						
In Situ	0.67 (0.57–0.77)	0.59 (0.51–0.68)	0.16 (0.09–0.27)	0.17 (0.10–0.30)	1.00 (0.85–1.19) *	0.73 (0.62–0.87)
Regional	2.28 (2.25–2.30)	2.18 (2.16–2.20)	2.23 (2.18–2.27)	2.27 (2.22–2.31)	2.05 (2.01–2.09)	2.01 (1.97–2.05)
Distant	5.74 (5.68–5.79)	4.48 (4.43–4.53)	6.61 (6.50–6.73)	5.18 (5.08–5.28)	5.10 (5.01–5.20)	3.95 (3.87–4.03)
Unstaged	3.68 (3.62–3.74)	2.32 (2.28–2.35)	3.79 (3.66–3.91)	2.51 (2.43–2.60)	2.98 (2.88–3.08)	2.02 (1.95–2.09)
**Grade Differentiation**						
Moderate	1.62 (1.59–1.65)	1.41 (1.38–1.43)	1.40 (1.36–1.44)	1.31 (1.28–1.35)	1.16 (1.11–1.20)	1.11 (1.07–1.15)
Poor	2.71 (2.66–2.77)	1.81 (1.78–1.84)	2.40 (2.33–2.46)	1.72 (1.68–1.77)	1.37 (1.32–1.42)	1.20 (1.16–1.25)
Undifferentiated	3.54 (3.46–3.61)	2.05 (2.00–2.09)	2.51 (2.37–2.65)	1.77 (1.68–1.88)	1.57 (1.46–1.68)	1.38 (1.28–1.48)
Unknown	3.61 (3.54–3.67)	1.67 (1.64–1.70)	3.52 (3.43–3.61)	1.56 (1.52–1.60)	2.05 (1.98–2.13)	1.18 (1.14–1.23)
**Surgery (Yes)**	0.273 (0.271–0.276)	0.452 (0.448–0.456)	0.253 (0.250–0.256)	0.465 (0.458–0.473)	0.292 (0.287–0.296)	0.397 (0.390–0.404)
**Radiotherapy (Yes)**	1.335 (1.328–1.343)	1.034 (1.028–1.040)	1.53 (1.52–1.55)	1.12 (1.11–1.13)	1.48 (1.46–1.49)	1.00 (0.99–1.02) *
**Chemotherapy (Yes)**	1.197 (1.190–1.204)	0.665 (0.660–0.669)	1.27 (1.25–1.28)	0.61 (0.60–0.62)	1.16 (1.15–1.17)	0.66 (0.65–0.67)

*p* < 0.05 relative to reference unless noted by * *p* ≥ 0.05. Reference categories: Gender (Male), Race (White), Detection Stage (Localized), Grade differentiation (Well), Surgery (No), Radiotherapy (No), and Chemotherapy (No). CI, confidence interval.

**Table 7:** Baseline demographics and clinical characteristics by histology for rectal cancers.

Rectal	All	Adenocarcinoma	Mucinous
***N* (%)**	194,109 (100)	119,273 (61.4)	8845 (4.6)
**Age (Years) (%)**			
0–14	10 (<0.1)	2 (<0.1)	0 (0)
15–29	1403 (0.7)	636 (0.5)	87 (1.0)
30–49	27,051 (13.9)	16,245 (13.6)	1367 (15.5)
50–69	97,233 (50.1)	57,992 (48.6)	4142 (46.8)
70–85	55,826 (28.8)	36,187 (30.3)	2711 (30.7)
>85	12,586 (6.5)	8211 (6.9)	538 (6.1)
**Mean (SD)**	63.7 (13.7)	64.4 (13.6)	63.8 (14.2)
**Gender (%)**			
Male	110,203 (56.8)	69,952 (58.6) *	5202 (58.8) *
Female	83,906 (43.2)	49,321 (41.4) *	3643 (41.2) *
**Race (%)**			
White	154,869 (79.8)	96,639 (81.0)	7245 (81.9)
Black	19,448 (10.0)	10,550 (8.8)	883 (10.0)
Other	19,792 (10.2)	12,084 (10.1)	717 (8.1)
**Detection Stage (%)**			
In Situ	9251 (4.8)	730 (0.6)	4 (<0.1)
Localized	78,378 (40.4)	37,686 (31.6)	2140 (24.2)
Regional	64,405 (33.2)	50,775 (42.6)	4654 (52.6)
Distant	30,903 (15.9)	23,972 (20.1)	1773 (20.0)
Unstaged	11,172 (5.8)	6110 (5.1)	274 (3.1)
**Grade Differentiation (%)**			
Well	17,774 (9.2)	7599 (6.4)	758 (8.6)
Moderate	108,345 (55.8)	82,503 (69.2)	4927 (55.7)
Poor	22,844 (11.8)	15,736 (13.2)	1617 (18.3)
Undifferentiated	1999 (1.0)	934 (0.8)	131 (1.5)
Unknown	43,147 (22.2)	12,501 (10.5)	1412 (16.0)
**Surgery (%)**			
Yes	156,833 (80.8)	94,549 (79.3)	7811 (88.3)
No	37,276 (19.2)	24,724 (20.7)	1034 (11.7)
**Radiotherapy (%)**			
Yes	71,720 (36.9)	55,224 (46.3)	4612 (52.1)
No	122,389 (63.1)	64,049 (53.7)	4233 (47.9)
**Chemotherapy (%)**			
Yes	86,677 (44.7)	67,533 (56.6)	5455 (61.7)
No	107,432 (55.3)	51,720 (43.4)	3390 (38.3)
**Incidence Rate (95% CI)**			
All	12.81 (12.75–12.87)	8.02 (7.97–8.06)	5.9 (5.7–6.0) **†**
Male	16.11 (16.01–16.21)	10.45 (10.37–10.53)	7.6 (7.4–7.8) **†**
Female	10.10 (10.03–10.17)	6.02 (5.96–6.07)	4.4 (4.3–4.6) **†**
**CSS % (95% CI)**			
1-year	86.4 (86.2–86.5)	85.3 (85.0–85.5) *	84.9 (84.0–85.8) *
2-year	78.4 (78.2–78.7)	76.0 (75.7–76.3)	72.2 (71.0–73.3)
5-year	64.8 (64.5–65.0)	59.8 (59.4–60.1)	53.1 (51.8–54.4)
10-year	56.8 (56.5–57.2)	50.7 (50.3–51.1)	44.1 (42.6–45.5)
Median (Months)	-	-	72.2
**RS % (95% CI)**			
1-year	85.2 (85.0–85.5)	84.1 (83.9–84.4) *	84.3 (83.1–85.3) *
2-year	77.6 (77.3–77.8)	75.1 (74.8–75.4)	71.7 (70.4–72.9)
5-year	64.5 (64.2–64.9)	59.5 (59.1–59.9)	53.5 (51.9–55.0)
10-year	56.5 (56.0–56.9)	50.2 (49.6–50.8)	43.3 (41.3–45.2)
Median (Months)	-	-	72.4

*p* < 0.05 for all comparisons between adenocarcinoma and mucinous adenocarcinoma, unless noted by * *p* ≥ 0.05. Incidence rates expressed per 100,000, except **†** (per 1 million). SD, standard deviation; CSS, cause-specific survival; RS, relative survival; CI, confidence interval.

**Table 8:** Derived univariate and multivariable Cox-proportional hazard ratios (HR) of mortality for rectal cancers.

Rectal	Mucinous vs. Non-Mucinous		Mucinous vs. Adenocarcinoma	
HR (95% CI)	Univariate	Multivariable	Univariate	Multivariable
**Mucinous Histology**	1.60 (1.55–1.65)	1.35 (1.31–1.39)	1.23 (1.19–1.27)	1.28 (1.24–1.32)
**Age (per 10 years)**	1.242 (1.235–1.250)	1.307 (1.298–1.315)	1.18 (1.17–1.19)	1.24 (1.23–1.25)
**Gender (Female)**	0.94 (0.92–0.95)	0.93 (0.91–0.94)	0.98 (0.96–0.997)	0.95 (0.93–0.96)
**Race**				
Black	1.16 (1.13–1.19)	1.19 (1.16–1.22)	1.39 (1.35–1.43)	1.27 (1.24–1.31)
Other	0.88 (0.86–0.91)	0.94 (0.91–0.96)	0.95 (0.93–0.98)	0.97 (0.94–1.00) *
**Detection Stage**				
In Situ	0.37 (0.34–0.40)	0.38 (0.35–0.42)	0.46 (0.36–0.58)	0.36 (0.28–0.46)
Regional	2.80 (2.73–2.86)	2.89 (2.82–2.96)	2.00 (1.95–2.05)	2.33 (2.27–2.40)
Distant	14.6 (14.2–14.9)	12.4 (12.1–12.8)	10.4 (10.1–10.7)	9.74 (9.45–10.0)
Unstaged	4.53 (4.37–4.69)	2.77 (2.67–2.88)	4.52 (4.33–4.71)	2.63 (2.52–2.75)
**Grade Differentiation**				
Moderate	1.70 (1.64–1.76)	1.25 (1.21–1.30)	1.14 (1.09–1.18)	1.07 (1.03–1.12)
Poor	3.01 (2.89–3.13)	1.88 (1.81–1.95)	1.86 (1.78–1.94)	1.60 (1.53–1.67)
Undifferentiated	3.09 (2.87–3.33)	2.16 (2.00–2.33)	1.87 (1.69–2.06)	1.60 (1.45–1.77)
Unknown	1.26 (1.21–1.31)	1.09 (1.05–1.13)	1.98 (1.89–2.08)	1.17 (1.12–1.23)
**Surgery (Yes)**	0.228 (0.224–0.232)	0.394 (0.386–0.402)	0.223 (0.219–0.228)	0.395 (0.386–0.404)
**Radiotherapy (Yes)**	1.28 (1.26–1.30)	1.09 (1.07–1.12)	0.82 (0.80–0.83)	1.04 (1.02–1.06)
**Chemotherapy (Yes)**	1.75 (1.72–1.78)	0.80 (0.78–0.82)	1.07 (1.05–1.09)	0.73 (0.71–0.74)

*p* < 0.05 relative to reference unless noted by * *p* ≥ 0.05. Reference categories: Gender (Male), Race (White), Detection Stage (Localized), Grade differentiation (Well), Surgery (No), Radiotherapy (No), and Chemotherapy (No). CI, confidence interval.

**Table 9:** Baseline demographics and clinical characteristics by histology for pancreatic cancers.

Pancreatic	All	Adenocarcinoma	Mucinous
***N* (%)**	160,539 (100)	93,923 (58.5)	5519 (3.5)
**Age (Years) (%)**			
0–14	61 (<0.1)	0 (0)	0 (0)
15–29	450 (0.3)	66 (0.1)	5 (0.1)
30–49	10,797 (6.7)	6062 (6.5)	437 (7.9)
50–69	68,769 (42.8)	44,493 (47.4)	2606 (47.2)
70–85	62,810 (39.1)	36,664 (39.0)	2188 (39.6)
>85	17,652 (11.0)	6638 (7.1)	283 (5.1)
**Mean (SD)**	68.9 (12.8)	67.8 (11.7)	66.9 (11.8)
**Gender (%)**			
Male	79,757 (49.7)	48,192 (51.3)	2670 (48.4)
Female	80,782 (50.3)	45,731 (48.7)	2849 (51.6)
**Race (%)**			
White	128,028 (79.7)	74,880 (79.7)	4449 (80.6)
Black	19,779 (12.3)	11,911 (12.7)	641 (11.6)
Other	12,732 (7.9)	7132 (7.6)	429 (7.8)
**Detection Stage (%)**			
In Situ	645 (0.4)	41 (<0.1)	8 (0.1)
Localized	14,669 (9.1)	6418 (6.8)	382 (6.9)
Regional	43,867 (27.3)	27,826 (29.6)	1357 (24.6)
Distant	84,626 (52.7)	55,075 (58.6)	3591 (65.1)
Unstaged	16,732 (10.4)	4563 (4.9)	181 (3.3)
**Grade Differentiation (%)**			
Well	8768 (5.5)	3675 (3.9)	528 (9.6)
Moderate	21,250 (13.2)	13,743 (14.6)	1080 (19.6)
Poor	22,061 (13.7)	14,872 (15.8)	713 (12.9)
Undifferentiated	1764 (1.1)	582 (0.6)	28 (0.5)
Unknown	106,696 (66.5)	61,051 (65.0)	3170 (57.4)
**Surgery (%)**			
Yes	32,963 (20.5)	15,671 (16.7)	1340 (24.3)
No	127,576 (79.5)	78,252 (83.3)	4179 (75.7)
**Radiotherapy (%)**			
Yes	23,323 (14.5)	16,224 (17.3)	866 (15.7)
No	137,216 (85.5)	77,699 (82.7)	4653 (84.3)
**Chemotherapy (%)**			
Yes	66,287 (41.3)	47,158 (50.2)	2670 (48.4)
No	94,252 (58.7)	46,765 (49.8)	2849 (51.6)
**Incidence Rate (95% CI)**			
All	12.23 (12.18–12.29)	6.97 (6.93–7.02)	3.8 (3.7–3.9) **†**
Male	13.89 (13.79–13.98)	8.04 (7.97–8.11)	4.2 (4.0–4.3) **†**
Female	10.87 (10.80–10.94)	6.10 (6.04–6.15)	3.6 (3.4–3.7) **†**
**CSS % (95% CI)**			
1-year	31.3 (31.0–31.6)	26.6 (26.3–26.9)	31.5 (30.0–32.9)
2-year	17.0 (16.8–17.2)	10.7 (10.5–11.0)	16.4 (15.2–17.6)
5-year	9.2 (9.0–9.4)	3.8 (3.6–3.9)	8.0 (7.1–9.0)
10-year	6.9 (6.7–7.1)	2.5 (2.4–2.7)	6.1 (5.1–7.1)
Median (Months)	5.9	5.6	6.3
**RS % (95% CI)**			
1-year	30.4 (30.2–30.7)	25.9 (25.6–26.2)	30.6 (29.2–32.0)
2-year	16.4 (16.1–16.6)	10.3 (10.1–10.6)	15.9 (14.7–17.1)
5-year	8.7 (8.5–8.9)	3.6 (3.4–3.7)	7.6 (6.7–8.6)
10-year	6.1 (5.9–6.3)	2.2 (2.1–2.4)	5.3 (4.3–6.5)
Median (Months)	5.7	5.4	6.1

*p* < 0.05 for all comparisons between adenocarcinoma and mucinous adenocarcinoma. Incidence rates expressed per 100,000, except **†** (per 1 million). SD, standard deviation; CSS, cause-specific survival; RS, relative survival; CI, confidence interval.

**Table 10:** Derived univariate and multivariable Cox-proportional hazard ratios (HR) of mortality for pancreatic cancers.

Pancreatic	Mucinous vs. Non-Mucinous		Mucinous vs. Adenocarcinoma	
HR (95% CI)	Univariate	Multivariable	Univariate	Multivariable
**Mucinous Histology**	1.02 (0.99–1.05) *	1.02 (0.99–1.06) *	0.85 (0.83–0.88)	0.87 (0.84–0.90)
**Age (per 10 years)**	1.215 (1.209–1.221)	1.170 (1.164–1.176)	1.121 (1.114–1.129)	1.090 (1.083–1.097)
**Gender (Female)**	1.01 (0.995–1.02) *	0.97 (0.96–0.98)	0.99 (0.98–1.01) *	0.96 (0.95–0.98)
**Race**				
Black	1.03 (1.01–1.05)	1.05 (1.03–1.07)	1.08 (1.06–1.10)	1.07 (1.05–1.09)
Other	0.93 (0.91–0.95)	0.95 (0.93–0.97)	0.98 (0.95–1.01) *	0.95 (0.92–0.98)
**Detection Stage**				
In Situ	0.11 (0.09–0.14)	0.13 (0.10–0.17)	0.11 (0.06–0.21)	0.12 (0.06–0.22)
Regional	1.55 (1.51–1.59)	1.70 (1.66–1.75)	1.11 (1.08–1.14)	1.34 (1.30–1.39)
Distant	3.01 (2.94–3.09)	2.64 (2.57–2.70)	2.26 (2.19–2.33)	2.28 (2.21–2.35)
Unstaged	2.46 (2.39–2.54)	1.53 (1.49–1.58)	1.63 (1.56–1.70)	1.26 (1.21–1.32)
**Grade Differentiation**				
Moderate	1.85 (1.79–1.92)	1.86 (1.80–1.92)	1.15 (1.11–1.20)	1.27 (1.22–1.32)
Poor	2.75 (2.66–2.85)	2.43 (2.35–2.51)	1.60 (1.54–1.66)	1.61 (1.55–1.67)
Undifferentiated	2.74 (2.57–2.92)	2.26 (2.12–2.41)	1.69 (1.53–1.86)	1.55 (1.41–1.72)
Unknown	3.11 (3.01–3.20)	1.86 (1.80–1.92)	1.83 (1.77–1.90)	1.35 (1.30–1.40)
**Surgery (Yes)**	0.343 (0.337–0.348)	0.453 (0.445–0.461)	0.42 (0.41–0.43)	0.52 (0.51–0.54)
**Radiotherapy (Yes)**	0.69 (0.68–0.70)	0.99 (0.98–1.01) *	0.58 (0.57–0.59)	0.93 (0.91–0.95)
**Chemotherapy (Yes)**	0.77 (0.76–0.78)	0.67 (0.66–0.68)	0.58 (0.57–0.59)	0.546 (0.537–0.554)

*p* < 0.05 relative to reference unless noted by * *p* ≥ 0.05. Reference categories: Gender (Male), Race (White), Detection Stage (Localized), Grade differentiation (Well), Surgery (No), Radiotherapy (No), and Chemotherapy (No). CI, confidence interval.

**Table 11:** Baseline demographics and clinical characteristics by histology for gastric cancers.

Gastric	All	Adenocarcinoma	Mucinous
***N* (%)**	106,972 (100)	65,218 (61.0)	2549 (2.4)
**Age (Years) (%)**			
0–14	38 (<0.1)	3 (<0.1)	0 (0)
15–29	858 (0.8)	338 (0.5)	12 (0.5)
30–49	12,374 (11.6)	5688 (8.7)	261 (10.2)
50–69	43,379 (40.6)	25,731 (39.5)	1010 (39.6)
70–85	39,088 (36.5)	25,902 (39.7)	1042 (40.9)
>85	11,235 (10.5)	7556 (11.6)	224 (8.8)
**Mean (SD)**	67.0 (14.4)	68.6 (13.7)	67.7 (13.5)
**Gender (%)**			
Male	64,729 (60.5)	42,788 (65.6) *	1675 (65.7) *
Female	42,243 (39.5)	22,430 (34.4) *	874 (34.3) *
**Race (%)**			
White	75,037 (70.1)	45,689 (70.1)	1824 (71.6)
Black	14,627 (13.7)	8467 (13.0)	376 (14.8)
Other	17,308 (16.2)	11,062 (17.0)	349 (13.7)
**Detection Stage (%)**			
In Situ	1021 (1.0)	475 (0.7)	0 (0)
Localized	27,653 (25.9)	14,922 (22.9)	443 (17.4)
Regional	28,402 (26.6)	18,989 (29.1)	930 (36.5)
Distant	38,065 (35.6)	24,333 (37.3)	995 (39.0)
Unstaged	11,831 (11.1)	6499 (10.0)	181 (7.1)
**Grade Differentiation (%)**			
Well	5762 (5.4)	2903 (4.5)	105 (4.1)
Moderate	21,817 (20.4)	18,465 (28.3)	748 (29.3)
Poor	52,910 (49.5)	34,003 (52.1)	1165 (45.7)
Undifferentiated	2524 (2.4)	1015 (1.6)	31 (1.2)
Unknown	23,959 (22.4)	8832 (13.5)	500 (19.6)
**Surgery (%)**			
Yes	56,058 (52.4)	33,371 (51.2)	1557 (61.1)
No	50,914 (47.6)	31,847 (48.8)	992 (38.9)
**Radiotherapy (%)**			
Yes	21,836 (20.4)	15,520 (23.8) *	631 (24.8) *
No	85,136 (79.6)	49,698 (76.2) *	1918 (75.2) *
**Chemotherapy (%)**			
Yes	42,224 (39.5)	26,979 (41.4) *	1080 (42.4) *
No	64,748 (60.5)	38,239 (58.6) *	1469 (57.6) *
**Incidence Rate (95% CI)**			
All	7.57 (7.53–7.62)	4.50 (4.47–4.54)	1.55 (1.49–1.62) **†**
Male	10.4 (10.3–10.5)	6.79 (6.72–6.86)	2.37 (2.25–2.50) **†**
Female	5.37 (5.32–5.43)	2.71 (2.68–2.75)	0.91 (0.85–0.98) **†**
**CSS % (95% CI)**			
1-year	55.7 (55.3–56.0)	52.9 (52.4–53.3) *	52.0 (49.6–54.4) *
2-year	41.7 (41.3–42.0)	37.1 (36.7–37.6) *	35.1 (32.8–37.5) *
5-year	31.1 (30.8–31.5)	25.8 (25.4–26.2)	23.2 (21.0–25.4)
10-year	27.0 (26.6–27.4)	22.1 (21.6–22.5)	18.7 (16.5–21.1)
Median (Months)	15.6	13.5	13.0
**RS % (95% CI)**			
1-year	53.7 (53.4–54.1)	51.0 (50.5–51.4) *	50.3 (47.9–52.7) *
2-year	39.8 (39.4–40.1)	35.3 (34.9–35.8) *	33.7 (31.4–36.1) *
5-year	29.1 (28.7–29.5)	24.0 (23.5–24.4)	21.6 (19.4–23.9)
10-year	24.1 (23.6–24.6)	19.3 (18.8–19.9)	16.4 (14.0–19.1)
Median (Months)	14.2	12.5	12.2

*p* < 0.05 for all comparisons between adenocarcinoma and mucinous adenocarcinoma, unless noted by * *p* ≥ 0.05. Incidence rates expressed per 100,000, except **†** (per 1 million). SD, standard deviation; CSS, cause-specific survival; RS, relative survival; CI, confidence interval.

**Table 12:** Derived univariate and multivariable Cox-proportional hazard ratios (HR) of mortality for gastric cancers.

Gastric	Mucinous vs. Non-Mucinous		Mucinous vs. Adenocarcinoma	
HR (95% CI)	Univariate	Multivariable	Univariate	Multivariable
**Mucinous Histology**	1.21 (1.16–1.28)	1.16 (1.10–1.22)	1.07 (1.02–1.13)	1.09 (1.04–1.15)
**Age (per 10 years)**	1.092 (1.085–1.098)	1.132 (1.125–1.139)	1.06 (1.05–1.07)	1.095 (1.086–1.103)
**Gender (Female)**	0.88 (0.87–0.90)	0.92 (0.91–0.94)	1.01 (0.99–1.03) *	0.97 (0.95–0.99)
**Race**				
Black	0.94 (0.92–0.96)	1.00 (0.98–1.03) *	1.01 (0.98–1.04) *	1.02 (0.99–1.05) *
Other	0.81 (0.79–0.83)	0.86 (0.84–0.88)	0.74 (0.72–0.76)	0.81 (0.79–0.84)
**Detection Stage**				
In Situ	0.40 (0.34–0.48)	0.38 (0.32–0.45)	0.36 (0.29–0.45)	0.30 (0.24–0.38)
Regional	2.79 (2.72–2.86)	3.01 (2.93–3.09)	2.12 (2.05–2.19)	2.58 (2.50–2.67)
Distant	7.13 (6.95–7.31)	5.60 (5.44–5.76)	5.70 (5.53–5.88)	4.91 (4.74–5.08)
Unstaged	3.68 (3.56–3.80)	2.22 (2.14–2.30)	3.78 (3.63–3.94)	2.22 (2.13–2.32)
**Grade Differentiation**				
Moderate	2.60 (2.46–2.75)	1.87 (1.77–1.97)	1.60 (1.51–1.70)	1.28 (1.21–1.36)
Poor	3.83 (3.64–4.04)	2.56 (2.43–2.70)	2.24 (2.11–2.37)	1.72 (1.63–1.83)
Undifferentiated	3.42 (3.19–3.67)	2.50 (2.32–2.68)	2.22 (2.02–2.44)	1.86 (1.70–2.05)
Unknown	2.47 (2.34–2.61)	1.61 (1.52–1.70)	2.41 (2.26–2.56)	1.43 (1.35–1.53)
**Surgery (Yes)**	0.316 (0.311–0.321)	0.41 (0.40–0.42)	0.316 (0.310–0.322)	0.42 (0.41–0.43)
**Radiotherapy (Yes)**	1.01 (0.99–1.03) *	1.01 (0.99–1.04) *	0.84 (0.82–0.86)	0.98 (0.95–1.00) *
**Chemotherapy (Yes)**	1.26 (1.24–1.28)	0.66 (0.65–0.68) *	1.05 (1.03–1.07)	0.62 (0.61–0.64)

*p* < 0.05 relative to reference unless noted by * *p* ≥ 0.05. Reference categories: Gender (Male), Race (White), Detection Stage (Localized), Grade differentiation (Well), Surgery (No), Radiotherapy (No), and Chemotherapy (No). CI, confidence interval.

**Table 13:** Baseline demographics and clinical characteristics by histology for appendiceal cancers.

Appendiceal	All	Carcinoid	Adenocarcinoma	Mucinous
***N* (%)**	11,456 (100)	4562 (39.8)	1761 (15.4)	3266 (28.5)
**Age (Years) (%)**				
0–14	152 (1.3)	151 (3.3)	0 (0)	1 (<0.1)
15–29	990 (8.6)	871 (19.1)	35 (2.0)	59 (1.8)
30–49	3052 (26.6)	1457 (31.9)	333 (18.9)	837 (25.6)
50–69	5114 (44.6)	1680 (36.8)	851 (48.3)	1651 (50.6)
70–85	1867 (16.3)	354 (7.8)	455 (25.8)	639 (19.6)
>85	281 (2.5)	49 (1.1)	87 (4.9)	79 (2.4)
**Mean (SD)**	53.9 (17.2)	45.8 (18.0)	61.4 (14.7)	58.0 (13.9)
**Gender (%)**				
Male	5230 (45.7)	1999 (43.8)	931 (52.9)	1465 (44.9)
Female	6226 (54.3)	2563 (56.2)	830 (47.1)	1801 (55.1)
**Race (%)**				
White	9608 (83.9)	3983 (87.3)	1394 (79.2)	2686 (82.2)
Black	1093 (9.5)	370 (8.1)	241 (13.7)	293 (9.0)
Other	755 (6.6)	209 (4.6)	126 (7.2)	287 (8.8)
**Detection Stage (%)**				
In Situ	131 (1.1)	1 (<0.1)	20 (1.1)	15 (0.5)
Localized	4774 (41.7)	2790 (61.2)	661 (37.5)	746 (22.8)
Regional	2620 (22.9)	1087 (23.8)	513 (29.1)	629 (19.3)
Distant	3420 (29.9)	381 (8.4)	527 (29.9)	1760 (53.9)
Unstaged	511 (4.5)	303 (6.6)	40 (2.3)	116 (3.6)
**Grade Differentiation (%)**				
Well	3514 (30.7)	1873 (41.1)	242 (13.7)	1093 (33.5)
Moderate	2743 (23.9)	427 (9.4)	904 (51.3)	1000 (30.6)
Poor	1408 (12.3)	257 (5.6)	370 (21.0)	287 (8.8)
Undifferentiated	176 (1.5)	43 (0.9)	16 (0.9)	49 (1.5)
Unknown	3615 (31.6)	1962 (43.0)	229 (13.0)	837 (25.6)
**Surgery (%)**				
Yes	10,772 (94.0)	4481 (98.2)	1639 (93.1)	2931 (89.7)
No	684 (6.0)	81 (1.8)	122 (6.9)	335 (10.3)
**Radiotherapy (%)**				
Yes	228 (2.0)	13 (0.3)	73 (4.1)	100 (3.1)
No	11,228 (98.0)	4549 (99.7)	1688 (95.9)	3166 (96.9)
**Chemotherapy (%)**				
Yes	3616 (31.6)	453 (9.9)	723 (41.1)	1615 (49.4)
No	7840 (68.4)	4109 (90.1)	1038 (58.9)	1651 (50.6)
**Incidence Rate (95% CI)**				
All	8.5 (8.3–8.6) **†**	3.5 (3.4–3.6) **†**	1.32 (1.26–1.38) **†**	2.34 (2.27–2.42) **†**
Male	8.2 (8.0–8.4) **†**	3.1 (2.9–3.2) **†**	1.53 (1.43–1.63) **†**	2.27 (2.16–2.40) **†**
Female	8.8 (8.6–9.1) **†**	3.9 (3.7–4.0) **†**	1.17 (1.09–1.24) **†**	2.43 (2.32–2.54) **†**
**CSS % (95% CI)**				
1-year	87.7 (86.7–88.5)	92.1 (90.2–93.6)	82.7 (80.4–84.7)	88.0 (86.4–89.4)
2-year	78.7 (77.6–79.8)	88.1 (85.9–90.0)	70.7 (68.1–73.2)	78.0 (76.0–79.8)
5-year	63.1 (61.7–64.5)	78.3 (75.3–81.0)	54.0 (50.9–57.0)	60.2 (57.7–62.2)
10-year	53.1 (51.3–54.9)	68.6 (63.2–73.4)	47.2 (43.8–50.5)	47.6 (44.7–50.6)
Median (Months)	-	-	82.1	102
**RS % (95% CI)**				
1-year	87.3 (86.2–88.2)	91.1 (88.9–92.9)	81.6 (79.2–83.8)	88.1 (86.3–89.6)
2-year	78.9 (77.6–80.1)	88.0 (85.3–90.2)	70.6 (67.7–73.3)	78.6 (76.4–80.7)
5-year	64.3 (62.5–66.0)	78.8 (74.5–82.4)	55.1 (51.4–58.6)	62.0 (59.0–64.8)
10-year	54.3 (51.7–56.8)	61.8 (54.2–68.5)	48.8 (44.5–52.9)	49.2 (45.2–53.2)
Median (Months)	-	-	107	114

*p* < 0.05 for all comparisons among carcinoid, adenocarcinoma, and mucinous adenocarcinoma. Incidence rates expressed per 100,000, except **†** (per 1 million). SD, standard deviation; CSS, cause-specific survival; RS, relative survival; CI, confidence interval.

**Table 14:** Derived univariate and multivariable Cox-proportional hazard ratios (HR) of mortality for appendiceal cancers.

Appendiceal	Mucinous vs. Non-Mucinous		Mucinous vs. Carcinoid		Mucinous vs. Adenocarcinoma	
HR (95% CI)	Univariate	Multivariable	Univariate	Multivariable	Univariate	Multivariable
**Mucinous Histology**	1.44 (1.34–1.55)	0.81 (0.74–0.87)	3.28 (2.94–3.65)	1.23 (1.08–1.40)	0.84 (0.77–0.93)	0.64 (0.57–0.71)
**Age (per 10 years)**	1.26 (1.23–1.29)	1.25 (1.22–1.29)	1.37 (1.32–1.41)	1.28 (1.23–1.33)	1.11 (1.08–1.15)	1.16 (1.12–1.20)
**Gender (Female)**	1.02 (0.95–1.10) *	0.92 (0.86–0.99)	0.94 (0.85–1.03) *	0.85 (0.77–0.94)	0.93 (0.85–1.01)	0.87 (0.79–0.96)
**Race**						
Black	1.29 (1.15–1.44)	1.38 (1.23–1.55)	1.40 (1.20–1.64)	1.46 (1.25–1.70)	1.24 (1.07–1.42)	1.29 (1.12–1.49)
Other	1.13 (0.98–1.30) *	0.95 (0.82–1.10)	1.20 (0.99–1.46) *	0.94 (0.77–1.15)	0.92 (0.77–1.11) *	0.90 (0.75–1.07) *
**Detection Stage**						
In Situ	0.40 (0.17–0.97)	0.31 (0.13–0.75)	1.18 (0.16–8.38) *	0.91 (0.13–6.48) *	0.60 (0.19–1.88) *	0.49 (0.16–1.53) *
Regional	2.92 (2.58–3.30)	2.34 (2.06–2.65)	2.83 (2.38–3.37)	2.29 (1.92–2.73)	2.30 (1.96–2.71)	2.06 (1.74–2.43)
Distant	9.24 (8.31–10.3)	6.81 (6.04–7.68)	9.84 (8.51–11.4)	6.04 (5.11–7.15)	4.76 (4.15–5.46)	5.07 (4.34–5.91)
Unstaged	2.94 (2.31–3.75)	2.43 (1.90–3.12)	3.08 (2.24–4.25)	2.38 (1.72–3.30)	2.26 (1.67–3.08)	2.28 (1.66–3.12)
**Grade Differentiation**						
Moderate	2.05 (1.82–2.31)	1.71 (1.51–1.93)	2.21 (1.90–2.58)	1.64 (1.40–1.92)	1.51 (1.32–1.73)	1.49 (1.30–1.71)
Poor	5.64 (5.01–6.36)	3.52 (3.10–4.00)	5.21 (4.41–6.16)	4.01 (3.37–4.76)	3.46 (2.99–4.02)	2.95 (2.52–3.45)
Undifferentiated	5.08 (4.00–6.45)	2.89 (2.27–3.68)	4.92 (3.51–6.90)	3.18 (2.26–4.47)	3.33 (2.33–4.77)	2.46 (1.71–3.52)
Unknown	1.83 (1.63–2.05)	1.78 (1.59–2.01)	1.52 (1.32–1.74)	1.84 (1.60–2.13)	2.19 (1.91–2.52)	1.68 (1.45–1.94)
**Surgery (Yes)**	0.25 (0.22–0.27)	0.47 (0.42–0.53)	0.24 (0.21–0.27)	0.55 (0.47–0.64)	0.32 (0.29–0.37)	0.50 (0.44–0.58)
**Radiotherapy (Yes)**	1.86 (1.56–2.22)	1.20 (1.00–1.44)	2.04 (1.57–2.64)	1.14 (0.87–1.49) *	1.20 (0.97–1.48) *	1.05 (0.84–1.30) *
**Chemotherapy (Yes)**	3.26 (3.03–3.50)	1.41 (1.29–1.53)	3.51 (3.18–3.87)	1.42 (1.27–1.59)	1.68 (1.53–1.84)	1.10 (0.99–1.21) *

*p* < 0.05 relative to reference unless noted by * *p* ≥ 0.05. Reference categories: Gender (Male), Race (White), Detection Stage (Localized), Grade differentiation (Well), Surgery (No), Radiotherapy (No), and Chemotherapy (No). CI, confidence interval.

**Table 15:** Baseline demographics and clinical characteristics by histology for gallbladder/biliary cancers.

Gallbladder/Biliary	All	Adenocarcinoma	Cholangiocarcinoma	Mucinous
***N* (%)**	41,289 (100)	23,923 (57.9)	7483 (18.1)	1225 (3.0)
**Age (Years) (%)**				
0–14	13 (<0.1)	1 (<0.1)	2 (<0.1)	0 (0)
15–29	120 (0.3)	59 (0.2)	17 (0.2)	4 (0.3)
30–49	2927 (7.1)	1674 (7.0)	477 (6.4)	94 (7.7)
50–69	16,142 (39.1)	9637 (40.3)	2962 (39.6)	538 (43.9)
70–85	16,720 (40.5)	9867 (41.2)	2989 (39.9)	479 (39.1)
>85	5367 (13.0)	2685 (11.2)	1036 (13.8)	110 (9.0)
**Mean (SD)**	69.7 (13.2)	69.4 (12.8)	69.9 (13.1)	68.3 (12.5)
**Gender (%)**				
Male	17,677 (42.8)	9858 (41.2)	3766 (50.3)	522 (42.6)
Female	23,612 (57.2)	14,065 (58.8)	3717 (49.7)	703 (57.4)
**Race (%)**				
White	32,074 (77.7)	18,724 (78.3)	5757 (76.9)	939 (76.7)
Black	3980 (9.6)	2335 (9.8)	631 (8.4)	116 (9.5)
Other	5235 (12.7)	2864 (12.0)	1095 (14.6)	170 (13.9)
**Detection Stage (%)**				
In Situ	926 (2.2)	389 (1.6)	2 (<0.1)	2 (0.2)
Localized	8653 (21.0)	5486 (22.9)	986 (13.2)	223 (18.2)
Regional	13,856 (33.6)	8713 (36.4)	2281 (30.5)	480 (39.2)
Distant	13,012 (31.5)	7539 (31.5)	2756 (36.8)	475 (38.8)
Unstaged	4842 (11.7)	1796 (7.5)	1458 (19.5)	45 (3.7)
**Grade Differentiation (%)**				
Well	3503 (8.5)	2341 (9.8)	268 (3.6)	144 (11.8)
Moderate	10,297 (24.9)	7690 (32.1)	941 (12.6)	446 (36.4)
Poor	9102 (22.0)	6190 (25.9)	915 (12.2)	262 (21.4)
Undifferentiated	612 (1.5)	210 (0.9)	43 (0.6)	12 (1.0)
Unknown	17,775 (43.1)	7492 (31.3)	5316 (71.0)	361 (29.5)
**Surgery (%)**				
Yes	21,334 (51.7)	14,545 (60.8)	1516 (20.3)	827 (67.5)
No	19,955 (48.3)	9378 (39.2)	5967 (79.7)	398 (32.5)
**Radiotherapy (%)**				
Yes	6274 (15.2)	3961 (16.6)	1175 (15.7)	223 (18.2)
No	35,015 (84.8)	19,962 (83.4)	6308 (84.3)	1002 (81.2)
**Chemotherapy (%)**				
Yes	12,898 (31.2)	7965 (33.3)	2711 (36.2)	417 (34.0)
No	28,391 (68.8)	15,958 (66.7)	4772 (63.8)	808 (66.0)
**Incidence Rate (95% CI)**				
All	2.99 (2.96–3.02)	1.70 (1.67–1.72)	6.2 (6.0–6.3) **†**	7.9 (7.5–8.4) **††**
Male	3.09 (3.04–3.13)	1.69 (1.65–1.72)	7.3 (7.1–7.5) **†**	7.9 (7.2–8.7) **††**
Female	2.94 (2.90–2.97)	1.72 (1.69–1.75)	5.3 (5.1–5.5) **†**	8.0 (7.4–8.6) **††**
**CSS % (95% CI)**				
1-year	47.9 (47.4–48.5)	51.0 (50.3–51.8)	37.0 (35.8–38.2)	50.2 (46.8–52.6)
2-year	32.3 (31.7–32.8)	34.7 (34.0–35.5)	18.6 (17.5–19.6)	33.9 (30.5–37.3)
5-year	20.7 (20.2–21.3)	22.1 (21.4–22.8)	8.6 (7.8–9.5)	21.7 (18.6–24.9)
10-year	17.3 (16.7–17.8)	18.4 (17.7–19.2)	6.5 (5.7–7.4)	18.7 (15.5–22.0)
Median (Months)	11.0	12.5	7.2	12.2
**RS % (95% CI)**				
1-year	45.9 (45.3–46.4)	49.1 (48.3–49.8)	35.1 (33.9–36.3)	49.0 (45.6–52.4)
2-year	30.2 (29.7–30.8)	32.6 (31.9–33.4)	17.1 (16.1–18.1)	33.0 (29.7–36.4)
5-year	18.5 (18.0–19.0)	19.8 (19.2–20.6)	7.4 (6.7–8.2)	19.8 (16.8–23.0)
10-year	14.5 (13.9–15.1)	15.5 (14.7–16.4)	4.9 (4.1–5.7)	16.7 (13.2–20.5)
Median (Months)	10.1	11.6	6.6	11.5

*p* < 0.05 for all comparisons among adenocarcinoma, cholangiocarcinoma, and mucinous adenocarcinoma. Incidence rates expressed per 100,000, except **†** (per 1 million) **††** (per 10 million). SD, standard deviation; CSS, cause-specific survival; RS, relative survival; CI, confidence interval.

**Table 16:** Derived univariate and multivariable Cox-proportional hazard ratios (HR) of mortality for gallbladder/biliary cancers.

Gallbladder/Biliary	Mucinous vs. Non-Mucinous		Mucinous vs. Adenocarcinoma		Mucinous vs. Cholangiocarcinoma	
HR (95% CI)	Univariate	Multivariable	Univariate	Multivariable	Univariate	Multivariable
**Mucinous Histology**	1.03 (0.96–1.11) *	1.08 (1.01–1.16)	1.07 (1.00–1.15) *	1.05 (0.97–1.12) *	0.68 (0.63–0.74)	0.98 (0.90–1.07) *
**Age (per 10 years)**	1.17 (1.16–1.18)	1.15 (1.14–1.16)	1.14 (1.13–1.16)	1.14 (1.13–1.16)	1.14 (1.12–1.16)	1.11 (1.08–1.13)
**Gender (Female)**	1.07 (1.04–1.09)	1.05 (1.03–1.08)	1.08 (1.05–1.12)	1.07 (1.04–1.11)	1.17 (1.11–1.24)	1.05 (1.00–1.11)
**Race**						
Black	1.04 (1.00–1.09) *	1.05 (1.01–1.10)	1.11 (1.05–1.17)	1.09 (1.03–1.15)	1.06 (0.96–1.16) *	1.02 (0.92–1.12) *
Other	0.92 (0.88–0.95)	0.94 (0.91–0.98)	0.92 (0.87–0.96)	0.95 (0.90–0.99)	0.93 (0.86–0.99)	0.92 (0.86–0.99)
**Detection Stage**						
In Situ	0.17 (0.14–0.20)	0.17 (0.14–0.21)	0.18 (0.13–0.24)	0.17 (0.13–0.23)	0.17 (0.02–1.18) *	0.23 (0.03–1.64) *
Regional	1.59 (1.53–1.65)	1.73 (1.66–1.80)	1.56 (1.49–1.64)	1.71 (1.63–1.79)	0.98 (0.90–1.07) *	1.33 (1.22–1.45)
Distant	3.99 (3.84–4.14)	3.28 (3.14–3.42)	4.03 (3.85–4.22)	3.43 (3.25–3.61)	2.14 (1.97–2.33)	2.23 (2.04–2.44)
Unstaged	2.72 (2.59–2.86)	1.44 (1.37–1.52)	2.75 (2.56–2.95)	1.46 (1.35–1.57)	1.53 (1.39–1.69)	1.10 (0.99–1.21) *
**Grade Differentiation**						
Moderate	1.41 (1.34–1.49)	1.30 (1.23–1.38)	1.31 (1.23–1.39)	1.24 (1.17–1.32)	1.05 (0.92–1.21) *	1.06 (0.92–1.22) *
Poor	2.32 (2.19–2.45)	1.93 (1.82–2.04)	2.10 (1.97–2.24)	1.83 (1.71–1.95)	1.53 (1.33–1.76)	1.38 (1.20–1.59)
Undifferentiated	2.25 (2.01–2.52)	1.86 (1.66–2.09)	1.99 (1.67–2.36)	1.79 (1.50–2.13)	1.42 (0.98–2.05)	0.94 (0.65–1.35) *
Unknown	2.76 (2.62–2.91)	1.46 (1.38–1.55)	2.69 (2.52–2.86)	1.47 (1.38–1.58)	2.09 (1.85–2.37)	1.12 (0.98–1.28) *
**Surgery (Yes)**	0.32 (0.31–0.33)	0.43 (0.42–0.45)	0.33 (0.31–0.34)	0.44 (0.43–0.46)	0.37 (0.35–0.40)	0.42 (0.39–0.46)
**Radiotherapy (Yes)**	0.82 (0.79–0.85)	1.02 (0.98–1.06) *	0.81 (0.78–0.85)	1.03 (0.99–1.08) *	0.65 (0.60–0.69)	0.96 (0.89–1.04) *
**Chemotherapy (Yes)**	1.05 (1.03–1.08)	0.74 (0.72–0.76)	1.05 (1.01–1.08)	0.72 (0.69–0.74)	0.76 (0.73–0.81)	0.67 (0.63–0.71)

*p* < 0.05 relative to reference unless noted by * *p* ≥ 0.05. Reference categories: Gender (Male), Race (White), Detection Stage (Localized), Grade differentiation (Well), Surgery (No), Radiotherapy (No), and Chemotherapy (No). CI, confidence interval.

**Table 17:** Baseline demographics and clinical characteristics by histology for small bowel cancers.

Appendiceal	All	Carcinoid	Adenocarcinoma	Mucinous
***N* (%)**	25,899 (100)	13,837 (53.4)	6111 (23.6)	679 (2.6)
**Age (Years) (%)**				
0–14	19 (0.1)	4 (<0.1)	0 (0)	1 (0.1)
15–29	248 (1.0)	113 (0.8)	42 (0.7)	3 (0.4)
30–49	3912 (15.1)	2091 (15.1)	794 (13.0)	91 (13.4)
50–69	12,530 (48.4)	7337 (53.0)	2533 (41.4)	319 (47.0)
70–85	7567 (29.2)	3725 (26.9)	2129 (34.8)	225 (33.1)
>85	1623 (6.3)	567 (4.2)	613 (10.0)	40 (5.9)
**Mean (SD)**	63.6 (14.1)	62.5 (13.1)	66.4 (14.3)	64.5 (13.8)
**Gender (%)**				
Male	13,517 (52.2)	7074 (51.1)	3244 (53.1)	370 (54.5)
Female	12,382 (47.8)	6763 (48.9)	2867 (46.6)	309 (45.5)
**Race (%)**				
White	20,207 (78.0)	11,066 (80.0)	4521 (74.0)	520 (76.6)
Black	4180 (16.1)	2305 (16.7)	1136 (18.6)	124 (18.3)
Other	1512 (5.8)	466 (3.4)	454 (7.4)	35 (5.2)
**Detection Stage (%)**				
In Situ	188 (0.7)	10 (0.1)	32 (0.5)	0 (0)
Localized	8057 (31.1)	4662 (33.7)	1237 (20.2)	135 (19.9)
Regional	8590 (33.2)	4980 (36.0)	2100 (34.4)	265 (39.0)
Distant	7177 (27.7)	3359 (24.3)	2227 (36.4)	249 (36.7)
Unstaged	1887 (7.3)	826 (6.0)	515 (8.4)	30 (4.4)
**Grade Differentiation (%)**				
Well	6349 (24.5)	5293 (38.3)	453 (7.4)	65 (9.6)
Moderate	4867 (18.8)	1235 (8.9)	2559 (41.9)	316 (46.5)
Poor	3101 (12.0)	185 (1.3)	1880 (30.8)	160 (23.6)
Undifferentiated	515 (2.0)	52 (0.4)	62 (1.0)	7 (1.0)
Unknown	11,067 (42.7)	7072 (51.1)	1157 (18.9)	131 (19.3)
**Surgery (%)**				
Yes	20,068 (77.5)	11,733 (84.8)	3671 (60.1)	514 (75.7)
No	5831 (22.5)	2104 (15.2)	2440 (39.9)	165 (24.3)
**Radiotherapy (%)**				
Yes	993 (3.8)	134 (1.0)	604 (9.9)	60 (8.8)
No	24,906 (96.2)	13,703 (99.0)	5507 (90.1)	619 (91.2)
**Chemotherapy (%)**				
Yes	5280 (20.4)	724 (5.2)	2452 (40.1)	298 (43.9)
No	20,619 (79.6)	13,113 (94.8)	3659 (59.9)	381 (56.1)
**Incidence Rate (95% CI)**				
All	2.11 (2.08–2.13)	1.12 (1.11–1.14)	5.2 (5.1–5.4) **†**	5.6 (5.2–6.0) **††**
Male	2.49 (2.45–2.53)	1.29 (1.26–1.32)	6.4 (6.2–6.6) **†**	6.8 (6.2–7.5) **††**
Female	1.81 (1.78–1.84)	0.99 (0.97–1.01)	4.3 (4.1–4.4) **†**	4.6 (4.1–5.1) **††**
**CSS % (95% CI)**				
1-year	80.9 (80.4–81.5)	94.9 (94.4–95.3)	56.1 (54.7–57.5)	63.2 (58.9–67.3)
2-year	74.5 (73.9–75.1)	92.4 (91.8–92.9)	41.8 (40.3–43.2)	45.1 (40.5–49.5)
5-year	64.8 (64.1–65.5)	85.0 (84.2–85.8)	27.4 (26.0–28.9)	32.6 (28.1–37.1)
10-year	56.1 (55.2–57.0)	74.7 (73.3–75.9)	23.1 (21.7–24.6)	27.6 (23.0–32.4)
Median (Months)	-	-	16.2	20.3
**RS % (95% CI)**				
1-year	78.7 (78.1–79.3)	93.2 (92.6–93.8)	53.7 (52.3–55.1)	60.9 (56.5–65.1)
2-year	72.5 (71.8–73.2)	91.0 (90.2–91.7)	39.6 (38.2–41.0)	43.4 (38.9–47.9)
5-year	63.4 (62.5–64.2)	84.3 (83.1–85.5)	26.1 (24.6–27.5)	30.7 (26.0–35.5)
10-year	53.2 (51.9–54.5)	71.8 (69.5–73.9)	21.4 (19.8–23.2)	27.0 (21.6–32.8)
Median (Months)	-	-	14.4	18.8

*p* < 0.05 for all comparisons among carcinoid, adenocarcinoma, and mucinous adenocarcinoma. Incidence rates expressed per 100,000, except **†** (per 1 million) **††** (per 10 million). SD, standard deviation; CSS, cause-specific survival; RS, relative survival; CI, confidence interval.

**Table 18:** Derived univariate and multivariable Cox-proportional hazard ratios (HR) of mortality for small bowel cancers.

Small Bowel	Mucinous vs. Non-Mucinous		Mucinous vs. Carcinoid		Mucinous vs. Adenocarcinoma	
HR (95% CI)	Univariate	Multivariable	Univariate	Multivariable	Univariate	Multivariable
**Mucinous Histology**	2.66 (2.40–2.95)	1.67 (1.51–1.86)	6.86 (6.15–7.65)	4.39 (3.79–5.09)	0.90 (0.81–1.00) *	1.04 (0.93–1.16) *
**Age (per 10 years)**	1.31 (1.29–1.34)	1.32 (1.30–1.35)	1.37 (1.32–1.41)	1.46 (1.41–1.51)	1.20 (1.17–1.23)	1.16 (1.13–1.19)
**Gender (Female)**	0.97 (0.93–1.02) *	0.94 (0.90–0.98)	1.06 (0.98–1.15) *	0.97 (0.89–1.05) *	1.02 (0.96–1.09) *	0.99 (0.93–1.06) *
**Race**						
Black	1.02 (0.96–1.08) *	1.09 (1.02–1.16)	0.79 (0.71–0.89)	0.96 (0.85–1.08) *	1.00 (0.92–1.08) *	1.00 (0.91–1.08) *
Other	1.15 (1.05–1.27)	0.99 (0.90–1.08) *	0.74 (0.58–0.95)	0.81 (0.62–1.04) *	0.97 (0.86–1.11) *	0.88 (0.77–0.99)
**Detection Stage**						
In Situ	1.09 (0.74–1.61) *	0.91 (0.62–1.34) *	1.83 (0.26–13.0) *	1.46 (0.20–10.4) *	0.93 (0.48–1.80) *	0.74 (0.38–1.43) *
Regional	2.20 (2.05–2.36)	2.06 (1.91–2.21)	2.74 (2.37–3.16)	2.92 (2.52–3.38)	1.83 (1.65–2.04)	2.04 (1.83–2.28)
Distant	5.91 (5.53–6.32)	4.63 (4.32–4.97)	9.43 (8.25–10.8)	9.05 (7.88–10.4)	5.32 (4.79–5.90)	4.64 (4.14–5.20)
Unstaged	3.35 (3.02–3.71)	1.96 (1.76–2.18)	2.89 (2.29–3.64)	2.12 (1.67–2.68)	4.01 (3.45–4.66)	2.10 (1.80–2.46)
**Grade Differentiation**						
Moderate	3.96 (3.63–4.32)	3.40 (3.12–3.72)	2.49 (2.16–2.88)	1.48 (1.27–1.73)	1.19 (1.04–1.37)	1.12 (0.97–1.29) *
Poor	8.14 (7.46–8.88)	5.70 (5.20–6.24)	8.86 (7.48–10.5)	2.70 (2.23–3.28)	1.77 (1.53–2.04)	1.53 (1.32–1.76)
Undifferentiated	5.95 (5.17–6.85)	5.11 (4.43–5.90)	9.13 (6.50–12.8)	3.44 (2.43–4.86)	1.86 (1.34–2.57)	1.74 (1.26–2.42)
Unknown	2.07 (1.90–2.24)	1.63 (1.50–1.77)	1.51 (1.36–1.68)	1.42 (1.28–1.58)	2.45 (2.11–2.85)	1.14 (0.98–1.33) *
**Surgery (Yes)**	0.28 (0.26–0.29)	0.35 (0.34–0.37)	0.42 (0.38–0.46)	0.49 (0.44–0.54)	0.27 (0.25–0.29)	0.40 (0.37–0.44)
**Radiotherapy (Yes)**	2.93 (2.71–3.18)	1.37 (1.26–1.49)	4.07 (3.33–4.99)	1.27 (1.03–1.57)	1.07 (0.97–1.18) *	1.09 (0.98–1.20) *
**Chemotherapy (Yes)**	2.69 (2.57–2.82)	1.30 (1.23–1.37)	4.73 (4.29–5.21)	1.78 (1.59–1.99)	0.97 (0.91–1.04) *	0.69 (0.64–0.74)

*p* < 0.05 relative to reference unless noted by * *p* ≥ 0.05. Reference categories: Gender (Male), Race (White), Detection Stage (Localized), Grade differentiation (Well), Surgery (No), Radiotherapy (No), and Chemotherapy (No). CI, confidence interval.

**Table 19:** Baseline demographics and clinical characteristics by histology for esophageal cancers.

Esophageal	All	Adenocarcinoma	Squamous Cell	Mucinous
***N* (%)**	59,989 (100)	30,860 (51.4)	20,634 (34.4)	853 (1.4)
**Age (Years) (%)**				
0–14	3 (<0.1)	0 (0)	3 (<0.1)	0 (0)
15–29	109 (0.2)	78 (0.3)	11 (0.1)	2 (0.2)
30–49	4597 (7.7)	2583 (8.4)	1356 (6.6)	79 (9.3)
50–69	30,938 (51.6)	16,299 (52.8)	10,583 (51.3)	454 (53.2)
70–85	20,281 (33.8)	10,002 (32.4)	7333 (35.5)	277 (32.5)
>85	4061 (6.8)	1898 (6.2)	1348 (6.5)	41 (4.8)
**Mean (SD)**	66.5 (12.0)	65.9 (12.1)	67.0 (11.6)	65.1 (12.3)
**Gender (%)**				
Male	46,488 (77.5)	26,477 (85.8)	13,442 (65.1)	744 (87.2)
Female	13,501 (22.5)	4383 (14.2)	7192 (34.9)	109 (12.8)
**Race (%)**				
White	49,399 (82.3)	29,242 (94.8)	12,696 (61.5)	816 (95.7)
Black	7273 (12.1)	830 (2.7)	5767 (27.9)	26 (3.0)
Other	3317 (5.5)	788 (2.6)	2171 (10.5)	11 (1.3)
**Detection Stage (%)**				
In Situ	906 (1.5)	398 (1.3)	256 (1.2)	0 (0)
Localized	12,352 (20.6)	6673 (21.6)	4332 (21.0)	170 (19.9)
Regional	18,347 (30.6)	9326 (30.2)	6954 (33.7)	324 (38.0)
Distant	20,768 (34.6)	11,556 (37.4)	6170 (29.9)	293 (34.3)
Unstaged	7616 (12.7)	2907 (9.4)	2922 (14.2)	66 (7.7)
**Grade Differentiation (%)**				
Well	2710 (4.5)	1595 (5.2)	961 (4.7)	39 (4.6)
Moderate	18,923 (31.5)	10,368 (33.6)	7985 (38.7)	238 (27.9)
Poor	24,233 (40.4)	12,995 (42.1)	7505 (36.4)	398 (46.7)
Undifferentiated	1068 (1.8)	392 (1.3)	185 (0.9)	9 (1.1)
Unknown	13,055 (21.8)	5510 (17.9)	3998 (19.4)	169 (19.8)
**Surgery (%)**				
Yes	18,433 (30.7)	11,160 (36.2)	4995 (24.2)	374 (43.8)
No	41,556 (69.3)	19,700 (63.8)	15,639 (75.8)	479 (56.2)
**Radiotherapy (%)**				
Yes	32,376 (54.0)	15,984 (51.8)	12,892 (62.5)	507 (59.4)
No	27,613 (46.0)	14,876 (48.2)	7742 (37.5)	346 (40.6)
**Chemotherapy (%)**				
Yes	34,006 (56.7)	18,169 (58.9)	11,887 (57.6)	554 (64.9)
No	25,983 (43.3)	12,691 (41.1)	8747 (42.4)	299 (35.1)
**Incidence Rate (95% CI)**				
All	4.40 (4.37–4.44)	2.27 (2.24–2.29)	1.47 (1.45–1.49)	5.8 (5.4–6.2) **††**
Male	7.58 (7.51–7.65)	4.33 (4.28–4.38)	2.10 (2.06–2.13)	11.1 (10.3–11.9) **††**
Female	1.82 (1.80–1.85)	0.59 (0.58–0.61)	0.96 (0.93–0.98)	1.5 (1.2–1.8) **††**
**CSS % (95% CI)**				
1-year	48.2 (47.7–48.6)	52.7 (52.0–53.3)	43.8 (42.9–44.6)	50.9 (46.7–54.7)
2-year	31.5 (31.0–31.9)	34.8 (34.2–35.5)	28.0 (27.2–28.8)	32.8 (29.1–36.6)
5-year	20.0 (19.6–20.4)	21.9 (21.3–22.5)	18.3 (17.6–19.0)	17.3 (14.2–20.7)
10-year	16.1 (15.7–16.6)	17.8 (17.2–18.4)	14.6 (13.8–15.3)	14.2 (11.0–17.8)
Median (Months)	11.3	13.2	9.7	12.3
**RS % (95% CI)**				
1-year	46.1 (45.6–46.5)	50.6 (50.0–51.2)	41.6 (40.8–42.5)	48.9 (45.0–52.8)
2-year	29.5 (29.1–30.0)	33.0 (32.4–33.6)	25.9 (25.2–26.7)	31.6 (27.9–35.3)
5-year	17.8 (17.4–18.2)	20.0 (19.4–20.6)	15.8 (15.1–16.5)	15.7 (12.7–18.9)
10-year	13.1 (12.7–13.6)	15.0 (14.4–15.7)	11.0 (10.2–11.8)	11.1 (8.1–14.6)
Median (Months)	10.5	12.3	9.1	11.7

*p* < 0.05 for all comparisons among adenocarcinoma, squamous cell carcinoma, and mucinous adenocarcinoma. Incidence rates expressed per 100,000, except **††** (per 10 million). SD, standard deviation; CSS, cause-specific survival; RS, relative survival; CI, confidence interval.

**Table 20:** Derived univariate and multivariable Cox-proportional hazard ratios (HR) of mortality for esophageal cancers.

Esophageal	Mucinous vs. Non-Mucinous		Mucinous vs. Adenocarcinoma		Mucinous vs. Squamous Cell	
HR (95% CI)	Univariate	Multivariable	Univariate	Multivariable	Univariate	Multivariable
**Mucinous Histology**	1.03 (0.95–1.11) *	1.15 (1.06–1.24)	1.12 (1.04–1.22)	1.19 (1.10–1.29)	0.92 (0.85–1.00) *	1.02 (0.94–1.11) *
**Age (per 10 years)**	1.116 (1.106–1.126)	1.10 (1.09–1.11)	1.12 (1.11–1.13)	1.11 (1.09–1.12)	1.09 (1.08–1.11)	1.07 (1.05–1.08)
**Gender (Female)**	1.00 (0.97–1.02) *	0.93 (0.90–0.95)	1.08 (1.03–1.12)	0.98 (0.94–1.02) *	0.85 (0.82–0.88)	0.85 (0.82–0.88)
**Race**						
Black	1.30 (1.26–1.34)	1.21 (1.17–1.24)	1.20 (1.11–1.31)	1.08 (0.99–1.17) *	1.23 (1.18–1.27)	1.16 (1.12–1.21)
Other	1.08 (1.04–1.13)	1.00 (0.96–1.05) *	1.05 (0.96–1.14) *	0.91 (0.83–0.99)	1.01 (0.95–1.07) *	0.96 (0.91–1.02) *
**Detection Stage**						
In Situ	0.29 (0.25–0.34)	0.27 (0.23–0.32)	0.29 (0.23–0.37)	0.27 (0.21–0.35)	0.35 (0.28–0.44)	0.28 (0.22–0.35)
Regional	1.59 (1.55–1.64)	1.85 (1.79–1.91)	1.91 (1.82–1.99)	2.17 (2.07–2.28)	1.23 (1.17–1.28)	1.41 (1.35–1.48)
Distant	3.46 (3.36–3.57)	3.25 (3.14–3.35)	4.57 (4.38–4.77)	3.97 (3.78–4.16)	2.35 (2.24–2.46)	2.34 (2.23–2.46)
Unstaged	2.30 (2.21–2.39)	1.76 (1.70–1.83)	2.94 (2.77–3.12)	2.04 (1.92–2.17)	1.65 (1.55–1.74)	1.40 (1.32–1.48)
**Grade Differentiation**						
Moderate	1.49 (1.41–1.57)	1.32 (1.24–1.39)	1.53 (1.42–1.65)	1.27 (1.18–1.37)	1.32 (1.21–1.43)	1.27 (1.17–1.39)
Poor	1.93 (1.83–2.04)	1.60 (1.52–1.69)	2.16 (2.01–2.32)	1.67 (1.55–1.80)	1.47 (1.35–1.60)	1.33 (1.22–1.45)
Undifferentiated	1.90 (1.74–2.07)	1.63 (1.49–1.78)	1.92 (1.68–2.20)	1.69 (1.48–1.94)	1.35 (1.12–1.63)	1.27 (1.05–1.53)
Unknown	1.40 (1.32–1.48)	1.19 (1.12–1.26)	1.37 (1.27–1.48)	1.15 (1.07–1.25)	1.27 (1.16–1.38)	1.17 (1.07–1.28)
**Surgery (Yes)**	0.39 (0.38–0.40)	0.49 (0.48–0.50)	0.32 (0.31–0.33)	0.44 (0.43–0.46)	0.55 (0.53–0.57)	0.58 (0.56–0.61)
**Radiotherapy (Yes)**	0.90 (0.88–0.92)	0.95 (0.92–0.97)	1.00 (0.98–1.03) *	1.03 (0.995–1.06) *	0.69 (0.67–0.71)	0.81 (0.77–0.84)
**Chemotherapy (Yes)**	0.87 (0.85–0.89)	0.59 (0.58–0.61)	1.06 (1.03–1.09)	0.60 (0.57–0.62)	0.63 (0.61–0.65)	0.57 (0.55–0.60)

*p* < 0.05 relative to reference unless noted by * *p* ≥ 0.05. Reference categories: Gender (Male), Race (White), Detection Stage (Localized), Grade differentiation (Well), Surgery (No), Radiotherapy (No), and Chemotherapy (No). CI, confidence interval.

**Table 21:** Baseline demographics and clinical characteristics by histology for prostate cancers.

Prostate	All	Adenocarcinoma	Mucinous
***N* (%)**	999,669 (100)	959,899 (96.0)	774 (0.1)
**Age (Years) (%)**			
0–14	44 (<0.1)	0 (0)	0 (0)
15–29	43 (<0.1)	10 (<0.1)	0 (0)
30–49	27,207 (2.7)	26,290 (2.7)	48 (6.2)
50–69	578,769 (57.9)	563,449 (58.7)	520 (67.2)
70–85	356,646 (35.7)	340,627 (35.5)	183 (23.6)
>85	36,960 (3.7)	29,523 (3.1)	23 (3.0)
**Mean (SD)**	67.1 (9.5)	66.8 (9.3)	64.0 (9.9)
**Race (%)**			
White	796,772 (79.7)	764,998 (79.7)	599 (77.4)
Black	148,696 (14.9)	143,025 (14.9)	126 (16.3)
Other	54,201 (5.4)	51,876 (5.4)	49 (6.3)
**Detection Stage (%)**			
In Situ	155 (<0.1)	75 (<0.1)	0 (0)
Localized/Regional	840,596 (84.1)	822,046 (85.6)	640 (82.7)
Distant	48,394 (4.8)	40,274 (4.2)	29 (3.7)
Unstaged	110,524 (11.1)	97,504 (10.2)	105 (13.6)
**Grade Differentiation (%)**			
Well	67,068 (6.7)	66,219 (6.9)	22 (2.8)
Moderate	503,255 (50.3)	494,704 (51.5)	323 (41.7)
Poor	365,431 (36.6)	358,221 (37.3)	372 (48.1)
Undifferentiated	2977 (0.3)	2541 (0.3)	9 (1.2)
Unknown	60,938 (6.1)	38,214 (4.0)	48 (6.2)
**Surgery (%)**			
Yes	439,779 (44.0)	426,321 (44.4)	530 (68.5)
No	559,890 (56.0)	533,578 (55.6)	244 (31.5)
**Radiotherapy (%)**			
Yes	343,522 (34.4)	336,965 (35.1)	189 (24.4)
No	656,147 (65.6)	622,934 (64.9)	585 (75.6)
**Chemotherapy (%)**			
Yes	8234 (0.8)	7086 (0.7)	14 (1.8)
No	991,435 (99.2)	952,813 (99.3)	760 (98.2)
**Incidence Rate (95% CI) ^**	141.2 (140.9–141.5)	134.1 (133.8–134.3)	8.6 (8.0–9.4) **††**
**CSS % (95% CI)**			
1-year	97.83 (97.79–97.86)	98.49 (98.45–98.52) *	98.3 (96.3–99.2) *
2-year	96.07 (96.03–96.12)	96.92 (96.87–96.96) *	96.0 (93.2–97.7) *
5-year	92.33 (92.26–92.39)	93.35 (93.28–93.42) *	91.1 (87.3–93.9) *
10-year	87.1 (87.0–87.2)	88.1 (88.0–88.2) *	89.1 (85.0–92.1) *
Median (Months)	-	-	-
**RS (Months) (95% CI)**			
1-year	99.00 (98.95–99.04)	99.70 (99.67–99.74) *	99.6 (98.7–99.99) *
2-year	98.37 (98.30–98.43)	99.35 (99.29–99.40) *	98.6 (90.9–99.8) *
5-year	97.4 (97.3–97.5)	98.7 (98.6–98.8) *	96.6 (87.0–99.2) *
10-year	95.8 (95.1–96.0)	97.2 (96.9–97.5) *	95.0 (85.7–98.3) *
Median (Months)	-	-	-

*p* < 0.05 for all comparisons between adenocarcinoma and mucinous adenocarcinoma, unless noted by * *p* ≥ 0.05. Incidence rates (**^** indicates calculated among male population) expressed per 100,000 except **††** (per 10 million). SD, standard deviation; CSS, cause-specific survival; RS, relative survival; CI, confidence interval.

**Table 22:** Derived univariate and multivariable Cox-proportional hazard ratios (HR) of mortality for prostate cancers.

Prostate	Mucinous vs. Non-Mucinous		Mucinous vs. Adenocarcinoma	
HR (95% CI)	Univariate	Multivariable	Univariate	Multivariable
**Mucinous Histology**	0.85 (0.68–1.06) *	0.95 (0.76–1.18) *	0.92 (0.74–1.14) *	0.97 (0.78–1.21) *
**Age (per 10 years)**	2.11 (2.09–2.12)	1.67 (1.65–1.68)	2.01 (1.99–2.02)	1.67 (1.66–1.69)
**Race**				
Black	1.27 (1.25–1.29)	1.28 (1.26–1.31)	1.30 (1.27–1.32)	1.31 (1.29–1.34)
Other	0.99 (0.97–1.02) *	0.77 (0.75–0.79)	0.98 (0.95–1.01) *	0.77 (0.74–0.79)
**Detection Stage**				
In Situ	1.21 (0.67–2.18) *	0.56 (0.31–1.01) *	1.17 (0.52–2.60) *	0.60 (0.27–1.31) *
Distant	27.4 (27.0–27.8)	13.6 (13.3–13.8)	26.7 (26.2–27.1)	14.2 (13.9–14.4)
Unstaged	3.02 (2.97–3.07)	2.62 (2.57–2.66)	2.66 (2.62–2.71)	2.55 (2.51–2.60)
**Grade Differentiation**				
Moderate	1.03 (0.99–1.08) *	1.37 (1.31–1.42)	1.03 (0.99–1.08) *	1.36 (1.30–1.41)
Poor	3.21 (3.09–3.34)	3.39 (3.26–3.53)	3.26 (3.13–3.39)	3.38 (3.24–3.52)
Undifferentiated	8.82 (8.22–9.47)	5.95 (5.54–6.38)	7.84 (7.26–8.47)	5.29 (4.89–5.71)
Unknown	8.87 (8.52–9.24)	3.87 (3.72–4.04)	6.50 (6.23–6.79)	3.37 (3.22–3.52)
**Surgery (Yes)**	0.486 (0.479–0.492)	0.72 (0.71–0.73)	0.524 (0.517–0.531)	0.73 (0.72–0.74)
**Radiotherapy (Yes)**	0.79 (0.78–0.80)	0.84 (0.83–0.86)	0.84 (0.83–0.85)	0.85 (0.84–0.86)
**Chemotherapy (Yes)**	7.21 (6.96–7.45)	2.30 (2.22–2.38)	6.97 (6.71–7.24)	2.24 (2.15–2.33)

*p* < 0.05 relative to reference unless noted by * *p* ≥ 0.05. Reference categories: Race (White), Detection Stage (Localized/Regional), Grade differentiation (Well), Surgery (No), Radiotherapy (No), and Chemotherapy (No). CI, confidence interval.

**Table 23:** Baseline demographics and clinical characteristics by histology for urinary bladder cancers.

Urinary Bladder	All	Transition Cell	Adenocarcinoma	Mucinous
***N* (%)**	252,104 (100)	237,005 (94.0)	1330 (0.5)	514 (0.2)
**Age (Years) (%)**				
0–14	92 (<0.1)	22 (<0.1)	0 (0)	0 (0)
15–29	844 (0.3)	767 (0.3)	16 (1.2)	10 (1.9)
30–49	14,211 (5.6)	13,146 (5.5)	167 (12.6)	128 (24.9)
50–69	101,475 (40.3)	96,290 (40.6)	528 (39.7)	242 (47.1)
70–85	107,689 (42.7)	101,623 (42.9)	471 (35.4)	111 (21.6)
>85	27,793 (11.0)	25,157 (10.6)	148 (11.1)	23 (4.5)
**Mean (SD)**	69.8 (12.5)	69.7 (12.3)	66.7 (15.1)	59.5 (14.6)
**Gender (%)**				
Male	189,668 (75.2)	180,006 (76.0)	846 (63.6)	322 (62.6)
Female	62,436 (24.8)	56,999 (24.0)	484 (36.4)	192 (37.4)
**Race (%)**				
White	226,912 (90.0)	214,042 (90.3)	1040 (78.2)	381 (74.1)
Black	13,717 (5.4)	12,259 (5.2)	194 (14.6)	96 (18.7)
Other	11,475 (4.6)	10,704 (4.5)	96 (7.2)	37 (7.2)
**Detection Stage (%)**				
In Situ	6454 (2.6)	6386 (2.7)	1 (0.1)	0 (0)
Localized	180,673 (71.7)	176,458 (74.5)	354 (26.6)	73 (14.2)
Regional	46,523 (18.5)	41,382 (17.5)	608 (45.7)	308 (59.9)
Distant	10,761 (4.3)	8002 (3.4)	294 (22.1)	108 (21.0)
Unstaged	7963 (3.1)	4777 (2.0)	73 (5.5)	25 (4.9)
**Grade Differentiation (%)**				
Well	32,449 (12.9)	31,563 (13.3)	64 (4.8)	80 (15.6)
Moderate	71,047 (28.2)	68,936 (29.1)	405 (30.5)	165 (32.1)
Poor	52,063 (20.7)	48,571 (20.5)	456 (34.3)	117 (22.8)
Undifferentiated	58,654 (23.3)	56,559 (23.9)	108 (8.1)	31 (6.0)
Unknown	37,891 (15.0)	31,376 (13.2)	297 (22.3)	121 (23.5)
**Surgery (%)**				
Yes	234,703 (93.1)	224,146 (94.6)	1137 (85.5)	485 (94.4)
No	17,401 (6.9)	12,859 (5.4)	193 (14.5)	29 (5.6)
**Radiotherapy (%)**				
Yes	11,546 (4.6)	9787 (4.1)	171 (12.9)	51 (9.9)
No	240,558 (95.4)	227,218 (95.9)	1159 (87.1)	463 (90.1)
**Chemotherapy (%)**				
Yes	42,371 (16.8)	39,255 (16.6)	307 (23.1)	114 (22.2)
No	209,733 (83.2)	197,750 (83.4)	1023 (76.9)	400 (77.8)
**Incidence Rate (95% CI)**				
All	20.45 (20.37–20.52)	19.04 (18.97–19.12)	1.07 (1.02–1.13) **†**	3.6 (3.3–4.0) **††**
Male	36.1 (35.9–36.2)	33.9 (33.7–34.0)	1.62 (1.52–1.72) **†**	4.9 (4.4–5.5) **††**
Female	8.82 (8.75–7.88)	7.98 (7.92–8.05)	0.67 (0.62–0.73) **†**	2.6 (2.2–3.0) **††**
**CSS % (95% CI)**				
1-year	91.0 (90.8–91.1)	92.6 (92.5–92.7)	73.5 (70.5–76.1)	82.1 (77.4–86.0)
2-year	86.2 (86.0–86.3)	88.1 (88.0–88.3)	63.6 (60.4–66.6)	69.5 (64.0–74.3)
5-year	80.0 (79.7–80.1)	82.0 (81.9–82.2)	49.4 (45.7–52.9)	51.0 (44.9–56.8)
10-year	74.0 (73.8–74.3)	76.1 (75.8–76.3)	41.6 (37.4–45.7)	39.6 (33.0–46.1)
Median (Months)	-	-	57.6	62.9
**RS % (95% CI)**				
1-year	90.1 (90.0–90.2)	92.0 (91.8–92.1)	70.2 (67.2–73.0)	82.1 (76.8–86.3)
2-year	85.2 (85.0–85.4)	87.4 (87.2–87.6)	59.4 (56.1–62.6)	69.7 (63.5–75.1)
5-year	78.6 (78.4–78.9)	81.0 (80.7–81.3)	43.3 (39.4–47.1)	49.5 (42.5–56.1)
10-year	71.4 (71.0–71.8)	73.7 (73.3–74.1)	36.5 (32.0–41.1)	38.1 (29.4–46.8)
Median (Months)	-	-	42.9	59.5

*p* < 0.05 for all comparisons among transition cell carcinoma, adenocarcinoma, and mucinous adenocarcinoma. Incidence rates expressed per 100,000, except **†** (per 1 million) **††** (per 10 million). SD, standard deviation; CSS, cause-specific survival; RS, relative survival; CI, confidence interval.

**Table 24:** Derived univariate and multivariable Cox-proportional hazard ratios (HR) of mortality for urinary bladder cancers.

	Mucinous vs. Non-Mucinous		Mucinous vs. Transition Cell		Mucinous vs. Adenocarcinoma	
HR (95% CI)	Univariate	Multivariable	Univariate	Multivariable	Univariate	Multivariable
**Mucinous Histology**	2.39 (2.10–2.71)	1.13 (0.99–1.28) *	2.63 (2.31–2.99)	1.26 (1.11–1.44)	0.86 (0.74–1.01) *	0.91 (0.77–1.07) *
**Age (per 10 years)**	1.49 (1.48–1.50)	1.47 (1.46–1.48)	1.52 (1.51–1.53)	1.50 (1.48–1.51)	1.20 (1.14–1.26)	1.22 (1.16–1.29)
**Gender (Female)**	1.27 (1.25–1.30)	1.09 (1.07–1.11)	1.19 (1.17–1.22)	1.05 (1.03–1.07)	1.29 (1.12–1.49)	1.11 (0.96–1.28)
**Race**						
Black	1.70 (1.65–1.75)	1.42 (1.37–1.46)	1.67 (1.61–1.73)	1.44 (1.39–1.49)	1.11 (0.92–1.33) *	1.24 (1.03–1.50)
Other	1.01 (0.96–1.05) *	0.88 (0.84–0.91)	1.00 (0.96–1.05) *	0.88 (0.84–0.92)	0.81 (0.61–1.09) *	1.06 (0.80–1.42) *
**Detection Stage**						
In Situ	0.23 (0.15–0.34)	0.24 (0.16–0.36)	0.26 (0.18–0.39)	0.27 (0.18–0.40)	-	-
Regional	6.56 (6.44–6.69)	4.60 (4.50–4.70)	6.63 (6.50–6.76)	4.37 (4.26–4.47)	2.07 (1.67–2.56)	2.17 (1.73–2.71)
Distant	26.9 (26.1–27.6)	19.5 (18.9–20.2)	28.1 (27.2–28.9)	19.4 (18.8–20.1)	7.19 (5.72–9.03)	6.92 (5.35–8.95)
Unstaged	4.17 (3.99–4.35)	2.82 (2.69–2.95)	2.99 (2.82–3.17)	2.28 (2.14–2.42)	2.07 (1.39–3.07)	1.50 (0.99–2.26) *
**Grade Differentiation**						
Moderate	1.58 (1.51–1.65)	1.42 (1.36–1.49)	1.56 (1.48–1.63)	1.42 (1.35–1.49)	1.37 (0.98–1.90) *	1.12 (0.80–1.57) *
Poor	6.23 (5.97–6.50)	2.68 (2.57–2.81)	6.28 (6.01–6.57)	2.83 (2.70–2.96)	2.89 (2.10–3.98)	2.16 (1.55–3.00)
Undifferentiated	6.70 (6.42–7.00)	2.63 (2.52–2.75)	7.13 (6.82–7.46)	2.85 (2.72–2.99)	1.97 (1.33–2.92)	1.66 (1.11–2.48)
Unknown	3.47 (3.31–3.64)	2.05 (1.95–2.15)	2.74 (2.60–2.88)	1.94 (1.84–2.04)	1.98 (1.42–2.77)	1.32 (0.94–1.86) *
**Surgery (Yes)**	0.49 (0.48–0.51)	0.64 (0.62–0.66)	0.64 (0.61–0.66)	0.68 (0.66–0.71)	0.35 (0.28–0.42)	0.57 (0.45–0.71)
**Radiotherapy (Yes)**	5.32 (5.18–5.45)	1.27 (1.24–1.31)	5.78 (5.62–5.95)	1.29 (1.25–1.33)	1.92 (1.59–2.31)	1.25 (1.02–1.54)
**Chemotherapy (Yes)**	2.19 (2.15–2.24)	0.97 (0.94–0.99)	2.25 (2.21–2.30)	0.98 (0.96–1.01) *	1.69 (1.45–1.97)	0.90 (0.75–1.07) *

*p* < 0.05 relative to reference unless noted by * *p* ≥ 0.05. Reference categories: Gender (Male), Race (White), Detection Stage (Localized), Grade differentiation (Well), Surgery (No), Radiotherapy (No), and Chemotherapy (No). CI, confidence interval.

**Table 25:** Baseline demographics and clinical characteristics by histology for anal cancers.

Anal	All	Squamous	Adenocarcinoma	Mucinous
***N* (%)**	31,057 (100)	23,440 (75.5)	1583 (5.1)	403 (1.3)
**Age (Years) (%)**				
0–14	3 (<0.1)	1 (<0.1)	0 (0)	0 (0)
15–29	728 (2.3)	636 (2.7)	8 (0.5)	3 (0.7)
30–49	8691 (28.0)	7358 (31.4)	189 (11.9)	56 (13.9)
50–69	15,023 (48.4)	11,452 (48.9)	645 (40.7)	161 (40.0)
70–85	5378 (17.3)	3341 (14.3)	547 (34.6)	139 (34.5)
>85	1234 (4.0)	652 (2.8)	194 (12.3)	44 (10.9)
**Mean (SD)**	57.5 (14.6)	55.8 (14.1)	67.4 (14.6)	66.5 (14.8)
**Gender (%)**				
Male	15,050 (48.5)	11,831 (50.5)	820 (51.8)	247 (61.3)
Female	16,007 (51.5)	11,609 (49.5)	763 (48.2)	156 (38.7)
**Race (%)**				
White	26,038 (83.8)	19,757 (84.3)	1248 (78.8)	273 (67.7)
Black	3835 (12.3)	2943 (12.6)	207 (13.1)	91 (22.6)
Other	1184 (3.8)	740 (3.2)	128 (8.1)	39 (9.7)
**Detection Stage (%)**				
In Situ	8782 (28.3)	8013 (34.2)	23 (1.5)	0 (0)
Localized	10,336 (33.3)	7302 (31.2)	528 (33.4)	128 (31.8)
Regional	7419 (23.9)	5368 (22.9)	544 (34.4)	195 (48.4)
Distant	2621 (8.4)	1575 (6.7)	322 (20.3)	56 (13.9)
Unstaged	1899 (6.1)	1182 (5.0)	166 (10.5)	25 (6.0)
**Grade Differentiation (%)**				
Well	2274 (7.3)	1855 (7.9)	138 (8.7)	69 (17.1)
Moderate	7359 (23.7)	5704 (24.3)	917 (57.9)	141 (35.0)
Poor	6296 (20.3)	4563 (19.5)	259 (16.4)	82 (20.3)
Undifferentiated	633 (2.0)	410 (1.7)	20 (1.3)	6 (1.5)
Unknown	14,495 (46.7)	10,908 (46.5)	249 (15.7)	105 (26.1)
**Surgery (%)**				
Yes	16,356 (52.7)	12,026 (51.3)	954 (60.3)	317 (78.7)
No	14,701 (47.3)	11,414 (48.7)	629 (39.7)	86 (21.3)
**Radiotherapy (%)**				
Yes	16,967 (54.6)	12,573 (53.6)	897 (56.7)	239 (59.3)
No	14,090 (45.4)	10,867 (46.4)	686 (43.3)	164 (40.7)
**Chemotherapy (%)**				
Yes	16,348 (52.6)	12,101 (51.6)	891 (56.3)	237 (58.8)
No	14,709 (79.3)	11,339 (48.4)	692 (43.7)	166 (41.2)
**Incidence Rate (95% CI)**				
All	1.73 (1.71–1.75)	1.21 (1.19–1.23)	1.27 (1.21–1.33) **†**	3.0 (2.7–3.3) **††**
Male	1.46 (1.43–1.49)	0.98 (0.96–1.01)	1.57 (1.48–1.68) **†**	3.9 (3.4–4.4) **††**
Female	1.96 (1.93–1.99)	1.40 (1.37–1.42)	1.03 (0.96–1.10) **†**	2.2 (1.9–2.6) **††**
**CSS % (95% CI)**				
1-year	87.8 (87.2–88.3)	89.0 (88.3–89.7)	81.2 (78.9–83.3)	88.9 (84.6–91.9)
2-year	79.0 (78.3–79.6)	81.1 (80.3–82.0)	66.7 (63.8–69.3)	76.7 (71.2–81.3)
5-year	68.7 (67.8–69.5)	72.6 (71.6–73.6)	47.7 (44.4–50.8)	53.8 (47.2–59.9)
10-year	63.5 (62.6–64.5)	67.8 (66.5–68.9)	39.6 (36.0–43.2)	45.7 (38.6–52.4)
Median (Months)	-	-	49.9	81.9
**RS % (95% CI)**				
1-year	86.4 (85.7–87.0)	87.5 (86.8–88.3)	80.6 (78.1–82.8)	86.4 (81.5–90.0)
2-year	76.7 (75.9–77.5)	79.0 (78.0–79.9)	64.9 (61.9–67.7)	73.7 (67.6–78.8)
5-year	64.3 (63.3–65.3)	68.0 (66.6–69.2)	42.5 (39.1–45.9)	50.9 (43.7–57.6)
10-year	56.4 (54.8–57.9)	61.4 (59.4–63.3)	28.1 (24.1–32.3)	36.0 (27.8–44.3)
Median (Months)	-	-	43.7	61.6

*p* < 0.05 for all comparisons among, squamous cell carcinoma, adenocarcinoma, and mucinous adenocarcinoma. Incidence rates expressed per 100,000, except **†** (per 1 million) **††** (per 1 million). SD, standard deviation; CSS, cause-specific survival; RS, relative survival; CI, confidence interval.

**Table 26:** Derived univariate and multivariable Cox-proportional hazard ratios (HR) of mortality for anal cancers.

Anal	Mucinous vs. Non-Mucinous		Mucinous vs. Squamous Cell		Mucinous vs. Adenocarcinoma	
HR (95% CI)	Univariate	Multivariable	Univariate	Multivariable	Univariate	Multivariable
**Mucinous Histology**	2.38 (2.04–2.77)	1.23 (1.06–1.44)	2.92 (2.51–3.41)	1.42 (1.21–1.67)	0.84 (0.71–0.99)	1.03 (0.86–1.23) *
**Age (per 10 years)**	1.36 (1.33–1.38)	1.23 (1.20–1.25)	1.33 (1.30–1.36)	1.19 (1.16–1.22)	1.16 (1.11–1.22)	1.20 (1.15–1.27)
**Gender (Female)**	1.04 (0.99–1.09) *	0.64 (0.61–0.67)	1.05 (0.98–1.11) *	0.60 (0.56–0.64)	1.05 (0.92–1.19) *	0.96 (0.84–1.10) *
**Race**						
Black	1.27 (1.19–1.37)	1.36 (1.27–1.47)	1.37 (1.26–1.50)	1.44 (1.32–1.57)	1.03 (0.86–1.25) *	1.01 (0.84–1.23) *
Other	1.13 (0.99–1.28) *	1.08 (0.95–1.22) *	0.88 (0.73–1.07) *	0.92 (0.76–1.11) *	1.05 (0.82–1.33) *	1.12 (0.88–1.43) *
**Detection Stage**						
In Situ	0.26 (0.24–0.29)	0.21 (0.18–0.23)	0.26 (0.23–0.29)	0.21 (0.18–0.25)	0.38 (0.12–1.19) *	0.47 (0.15–1.50) *
Regional	2.29 (2.15–2.45)	2.46 (2.31–2.63)	2.22 (2.05–2.40)	2.30 (2.12–2.50)	1.77 (1.48–2.11)	1.99 (1.65–2.40)
Distant	6.43 (5.99–6.90)	6.81 (6.32–7.33)	5.79 (5.29–6.33)	5.87 (5.34–6.46)	5.32 (4.41–6.42)	5.40 (4.40–6.62)
Unstaged	2.58 (2.34–2.83)	2.19 (1.99–2.42)	2.40 (2.13–2.71)	2.09 (1.85–2.37)	2.89 (2.25–3.71)	1.93 (1.48–2.51)
**Grade Differentiation**						
Moderate	1.55 (1.40–1.72)	1.43 (1.29–1.59)	1.43 (1.27–1.60)	1.37 (1.22–1.54)	1.56 (1.21–2.02)	1.37 (1.06–1.78)
Poor	1.57 (1.42–1.75)	1.49 (1.33–1.66)	1.39 (1.24–1.57)	1.39 (1.23–1.57)	2.25 (1.70–2.98)	1.90 (1.43–2.52)
Undifferentiated	1.31 (1.08–1.58)	2.19 (1.81–2.66)	0.54 (0.39–0.75)	1.32 (0.95–1.84) *	2.18 (1.20–3.95)	2.51 (1.38–4.56)
Unknown	0.74 (0.67–0.82)	1.31 (1.18–1.46)	0.53 (0.47–0.60)	1.14 (1.00–1.29) *	1.48 (1.11–1.98)	1.24 (0.92–1.67) *
**Surgery (Yes)**	0.58 (0.55–0.61)	0.81 (0.77–0.86)	0.53 (0.50–0.56)	0.79 (0.73–0.84)	0.36 (0.31–0.41)	0.45 (0.39–0.53)
**Radiotherapy (Yes)**	1.80 (1.70–1.89)	0.83 (0.77–0.90)	2.40 (2.24–2.57)	0.91 (0.82–1.02) *	0.92 (0.80–1.05) *	1.01 (0.84–1.21) *
**Chemotherapy (Yes)**	1.68 (1.59–1.77)	0.66 (0.61–0.72)	2.07 (1.93–2.21)	0.65 (0.59–0.72)	0.95 (0.83–1.09) *	0.72 (0.59–0.88)

*p* < 0.05 relative to reference unless noted by * *p* ≥ 0.05. Reference categories: Gender (Male), Race (White), Detection Stage (Localized), Grade differentiation (Well), Surgery (No), Radiotherapy (No), and Chemotherapy (No). CI, confidence interval.

## Supplementary Materials

The following are available online at https://www.mdpi.com/2072-6694/12/11/3193/s1, Table S1: Baseline demographics and clinical characteristics by histology for gynecological cancers, Table S2: Derived univariate and multivariable Cox-proportional hazard ratios (HR) of mortality for gynecological cancers, Table S3: Exclusion criteria and counts of all cases and mucinous cases, Table S4. Count of all cases and mucinous cases by SEER cancer registry for all included cases, Table S5: Variables in analysis. Table S6: Breakdown of mucinous cases in SEER (1975–2016), both analyzed and not analyzed.
