# Supplementary material for: Epidemiology of Mucinous Adenocarcinomas
Source: Cancers (Basel). 2020 Oct 30;12(11):3193. doi: 10.3390/cancers12113193 (PMC7692300; doi:10.3390/cancers12113193)
Supplement: Supplementary file 1 [file cancers-12-03193-s001.pdf]

cancers MDPI

Supplementary Material

Epidemiology of Mucinous Adenocarcinomas

Matthew G.K. Benesch and Alexander Mathieson

Table S1. Baseline demographics and clinical characteristics by histology for gynecological cancers.

Cancer Type	Ovarian		Uterine		Cervical	
	**All**	**Mucinous**	**All**	**Mucinous**	**All**	**Mucinous**
***N* (%)**	104,705 (100)	3862 (3.7)	202,098 (100)	1958 (1.0)	67,338 (100)	1094 (1.6)
**Age (Years) (%)**						
0-14	645 (0.6)	5 (0.1)	9 (<0.1)	0 (0)	19 (<0.1)	0 (0)
15-29	3906 (3.7)	267 (6.9)	1181 (0.6)	2 (0.1)	4260 (6.3)	37 (3.4)
30-49	20,498 (19.6)	1050 (27.2)	28,853 (14.3)	210 (10.7)	32,896 (48.9)	590 (53.9)
50-69	45,891 (43.8)	1639 (42.4)	117,238 (58.0)	1197 (61.1)	22,046 (32.7)	357 (32.6)
70-85	27,087 (25.9)	746 (19.3)	47,194 (23.4)	480 (24.5)	6709 (10.0)	95 (8.7)
>85	6678 (6.4)	155 (4.0)	7623 (3.8)	69 (3.5)	1408 (2.1)	15 (1.4)
**Mean (SD)**	60.7 (16.6)	56.1 (16.8)	62.0 (12.5)	63.0 (11.4)	49.7 (15.2)	49.2 (13.9)
**Race (%)**						
White	87,315 (83.4)	3093 (80.1)	166,021 (82.1)	1662 (84.9)	50,809 (75.5)	868 (79.3)
Black	8499 (8.1)	358 (9.3)	19,129 (9.5)	107 (5.5)	9439 (14.0)	69 (6.3)
Other	8891 (8.5)	411 (10.6)	16,948 (8.4)	189 (9.7)	7090 (10.5)	157 (14.4)
**Detection Stage (%)**						
In Situ	621 (0.6)	39 (1.0)	2357 (1.2)	18 (0.9)	0 (0)	0 (0)
Localized	23,494 (22.4)	1846 (47.8)	135,970 (67.3)	1420 (72.5)	32,282 (47.9)	549 (50.2)
Regional	9969 (9.5)	283 (7.3)	35,923 (17.8)	331 (16.9)	24,136 (35.8)	349 (31.9)
Distant	64,289 (61.4)	1572 (40.7)	19,757 (9.8)	116 (5.9)	7651 (11.4)	170 (15.5)
Unstaged	6332 (5.0)	122 (3.2)	8091 (4.0)	73 (3.7)	3269 (4.9)	26 (2.4)
**Grade Differentiation (%)**						
Well	7167 (6.8)	1012 (26.2)	69,707 (34.5)	991 (50.6)	6201 (9.2)	320 (29.3)
Moderate	12,908 (12.3)	1041 (27.0)	49,081 (24.3)	586 (29.9)	18,961 (28.2)	413 (37.8)
Poor	30,025 (28.7)	527 (13.6)	32,769 (16.2)	124 (6.3)	19,051 (28.3)	174 (15.9)

Undifferentiated	12,532 (12.0)	95 (2.5)	11,724 (5.8)	15 (0.8)	1657 (2.5)	20 (1.8)
Unknown	42,073 (40.2)	1187 (30.7)	38,817 (19.2)	242 (12.4)	21,468 (31.9)	167 (15.3)
**Surgery (%)**						
Yes	82,018 (78.3)	3322 (86.0)	185,246 (91.7)	1832 (93.6)	41,230 (61.2)	874 (79.9)
No	22,687 (21.7)	540 (14.0)	16,852 (8.3)	126 (6.4)	26,108 (38.8)	220 (20.1)
**Radiotherapy (%)**						
Yes	1567 (1.5)	59 (1.5)	50,947 (25.2)	453 (23.1)	34,818 (51.7)	543 (49.6)
No	103,138 (98.5)	3803 (98.5)	151,151 (74.8)	1505 (76.9)	32,520 (48.3)	551 (50.4)
**Chemotherapy (%)**						
Yes	63,158 (60.3)	1743 (45.1)	31,540 (15.6)	232 (11.8)	26,260 (39.0)	447 (40.9)
No	41,547 (39.7)	2119 (54.9)	170,558 (84.4)	1726 (88.2)	41,078 (61.0)	647 (59.1)
**Incidence Rate (95% CI)^**	12.5 (12.4-12.6)	4.8 (4.6-5.0)**†**	24.8 (24.7-24.9)	2.0 (1.9-2.1)**†**	8.02 (7.96-8.09)	1.4 (1.3-1.5)**†**
**CSS % (95% CI)**						
1-year	72.8 (72.5-73.2)	68.5 (66.4-70.5)	90.6 (90.4-90.8)	95.0 (96.1-93.4)	78.7 (78.1-79.3)	79.8 (74.3-84.3)
2-year	70.0 (60.6-61.3)	60.5 (58.3-62.6)	85.2 (85.0-85.4)	92.3 (90.4-93.9)	68.0 (67.3-68.7)	67.2 (61.2-72.5)
5-year	40.6 (40.1-40.9)	51.8 (49.5-54.0)	78.0 (77.8-78.3)	87.1 (84.7-89.3)	57.5 (56.8-58.2)	51.8 (45.5-57.7)
10-year	30.4 (30.0-30.8)	46.8 (44.3-49.4)	74.4 (74.1-74.7)	83.8 (80.8-86.3)	52.4 (51.6-53.2)	39.9 (33.0-46.7)
Median (Months)	39.2	77.4	-	-	-	64.9
**RS % (95% CI)**						
1-year	72.0 (71.7-72.3)	67.1 (65.0-69.1)	90.2 (90.0-90.4)	95.0 (93.0-96.4)	77.0 (76.4-77.6)	80.4 (74.5-85.0)
2-year	60.6 (60.2-60.9)	59.5 (57.2-61.6)	85.0 (84.8-85.3)	91.8 (89.3-93.7)	65.6 (64.9-66.3)	67.7 (61.2-73.3)
5-year	40.6 (40.2-41.0)	50.9 (48.3-53.4)	78.6 (78.3-78.9)	87.9 (84.6-90.6)	53.8 (53.0-54.6)	53.2 (45.9-59.9)
10-year	30.7 (30.2-31.2)	43.9 (40.6-47.2)	75.4 (74.8-75.9)	85.6 (81.6-88.8)	46.6 (45.5-47.6)	38.3 (31.0-45.5)
Median (Months)	38.9	67.3	-	-	85.7	75.5

Incidence rates (**^** indicates calculated among female population) expressed per 100,000, except **†** (per 1 million). SD, standard deviation; CSS, cause-specific survival; RS, relative survival; CI, confidence interval.

Table S2. Derived univariate and multivariable Cox-proportional hazard ratios (HR) of mortality for gynecological cancers.

Cancer Type	Ovarian		Uterine		Cervical	
	Mucinous vs. Non-Mucinous		Mucinous vs. Non-Mucinous		Mucinous vs. Non-Mucinous	
**HR (95% CI)**	**Univariate**	**Multivariable**	**Univariate**	**Multivariable**	**Univariate**	**Multivariable**
**Mucinous Histology**	0.75 (0.71-0.79)	1.52 (1.44-1.61)	0.58 (0.51-0.67)	0.90 (0.79-1.03)*	1.23 (1.10-1.37)	1.49 (1.33-1.66)
**Age (per 10 years)**	1.52 (1.51-1.53)	1.29 (1.28-1.30)	1.48 (1.47-1.50)	1.33 (1.32-1.34)	1.37 (1.36-1.38)	1.17 (1.15-1.18)
**Race**						
Black	1.17 (1.13-1.20)	1.19 (1.16-1.23)	2.43 (2.36-2.50)	1.45 (1.41-1.49)	1.47 (1.42-1.53)	1.21 (1.16-1.25)
Other	0.72 (0.70-0.75)	0.94 (0.91-0.97)	0.95 (0.91-0.99)	0.98 (0.94-1.02)*	0.96 (0.91-1.01)*	0.87 (0.83-0.91)
**Detection Stage**						
In Situ	0.34 (0.22-0.54)	0.34 (0.22-0.55)	0.35 (0.28-0.45)	0.31 (0.24-0.39)	-	-
Regional	4.70 (4.45-4.97)	3.74 (3.53-3.96)	5.08 (4.94-5.23)	3.55 (3.45-3.66)	6.32 (6.05-6.60)	3.99 (3.79-4.21)
Distant	13.2 (12.6-13.7)	9.14 (8.73-9.57)	19.5 (19.0-20.0)	9.13 (8.83-9.44)	22.0 (21.0-23.1)	13.4 (12.7-14.2)
Unstaged	13.0 (12.3-13.7)	5.24 (4.95-5.56)	6.65 (6.35-6.96)	2.30 (2.18-2.42)	5.68 (5.30-6.10)	3.55 (3.29-3.82)
**Grade Differentiation**						
Moderate	2.86 (2.69-3.05)	1.80 (1.69-1.92)	2.82 (2.70-2.95)	2.02 (1.93-2.11)	2.24 (2.08-2.42)	1.36 (1.26-1.47)
Poor	5.17 (4.87-5.49)	2.16 (2.04-2.30)	11.1 (10.6-11.5)	4.77 (4.58-4.98)	3.69 (3.42-3.97)	1.77 (1.64-1.91)
Undifferentiated	4.69 (4.41-5.00)	2.06 (1.94-2.20)	16.9 (16.1-17.7)	6.15 (5.86-6.45)	4.69 (4.23-5.20)	2.24 (2.02-2.49)
Unknown	4.43 (4.17-4.70)	1.87 (1.76-1.99)	7.18 (6.88-7.48)	3.57 (3.42-3.73)	2.02 (1.87-2.18)	1.29 (1.20-1.40)
**Surgery (Yes)**	0.213 (0.209-0.218)	0.36 (0.35-0.37)	0.169 (0.165-0.174)	0.31 (0.30-0.32)	0.214 (0.207-0.221)	0.47 (0.46-0.49)
**Radiotherapy (Yes)**	1.34 (1.26-1.43)	1.18 (1.11-1.26)	1.60 (1.57-1.64)	0.87 (0.85-0.89)	3.21 (3.10-3.31)	1.06 (1.01-1.10)
**Chemotherapy (Yes)**	1.64 (1.61-1.67)	0.91 (0.89-0.93)	4.46 (4.36-4.56)	1.07 (1.04-1.10)	2.62 (2.55-2.70)	0.81 (0.78-0.84)

$p < 0.05$ relative to reference unless noted by * $p \geq 0.05$. Reference categories: Race (White), Detection Stage (Localized), Grade differentiation (Well), Surgery (No), Radiotherapy (No), and Chemotherapy (No). CI, confidence interval.

cancers MDPI

Table S3. Exclusion criteria and counts of all cases and mucinous cases.

Data Processing Scheme	Count (All)	Count (Mucinous)
Initial count of all non-blood borne cancer cases.	9,608,247	169,595
Drop if year diagnosis ≤1991 (year_dx≤1991).	8,229,651	136,446
Drop if cancer is not patient's primary or first (seq_num≥2).	6,587,624	108,351
Drop if cause-specific death classification unknown/missing (vsrtsadx = 8).	6,545,754	107,538
Drop if survival months is unknown (srv_time_mon=9999).	6,468,886	107,383
Drop if age_dx is unknown (age_dx=999).	6,467,693	107,381
Drop if race unknown (race1v=99).	6,387,182	107,113
Drop if surgery is unknown (surgprif, ss_surg=99).	6,350,139	106,968
Drop if any variable in Table 3 missing data.	6,350,139	106,968

Table S4. Count of all cases and mucinous cases by SEER cancer registry for all included cases.

SEER Registry	SEER #	Years of Diagnosis	Count (All)	Count (Mucinous)
San Francisco-Oakland	SEER 9	1975-2016	389,446	6832
Connecticut	SEER 9	1975-2016	385,805	6762
Metropolitan Detroit	SEER 9	1975-2016	425,465	7383
Hawaii	SEER 9	1975-2016	118,685	1826
Iowa	SEER 9	1975-2016	314,191	6221
New Mexico	SEER 9	1975-2016	156,830	2553
Seattle-Puget Sound	SEER 9	1975-2016	418,995	6236
Utah	SEER 9	1975-2016	161,122	2675
Metropolitan Atlanta	SEER 9	1975-2016	239,639	3437
Alaska	SEER 13	1992-2016	7,496	134
San Jose-Monterey	SEER 13	1992-2016	190,550	3314
Los Angeles	SEER 13	1992-2016	719,362	14,588
Rural Georgia	SEER 13	1992-2016	13,136	238
Greater California	SEER 18	2000-2016	1,146,228	17,715
Kentucky	SEER 18	2000-2016	315,625	5182
Louisiana	SEER 18	2000-2016	310,603	5318
New Jersey	SEER 18	2000-2016	646,404	11,115
Greater Georgia	SEER 18	2000-2016	390,557	5439
**TOTAL**			6,350,139	106,968

Table S5. Variables in analysis. Categorization reflects final variable composition.

Variable (SEER Variable)	Variable Name/Description per SEER	Categorization
**Exposure**		
Histology (ICD-0-3)	848x/x (Mucinous Adenocarcinoma)<br>814x/x (Adenocarcinoma, NOS)<br>850x/x (Ductal Carcinoma) (Breast)<br>852x/x (Lobular and other Ductal Ca) (Breast)<br>807x/x (Squamous Cell Carcinoma, NOS)<br>816x/x (Cholangiocarcinoma) (Gallbladder/Biliary)<br>824x/x (Carcinoid Tumor, Malignant)	Mucinous – 848x<br>Adenocarcinoma - 814x<br>Ductal – 850x<br>Lobular – 852x<br>Squamous Cell – 807x<br>Cholangio. – 816x<br>Carcinoid – 824x

	812x/x, 813x/x (Transitional Cell Carcinoma, NOS & Papillary Transitional Cell Carcinoma)	Transition Cell – 812x,813x
	**Outcome**	
Mortality (vsrtsadx)	vsrtsadx (SEER cause-specific death classification) 0 (Alive or dead of other cause), 1 (Dead), 8 (Dead – missing/unknown cause of death), 9 (N/A not first tumor)	Alive/N/A – 0,9<br>Dead – 1
(srv_time_mon)	srv_time_mon (Survival months) 0-9998 (in months), 9999 (Unknown)	Months (0-9998)
	**Co-variables**	
Age (age_dx)	000-130 (Actual age of diagnosis in years), 999 (Unknown)	-
Gender (sex)	1 (Male), 2 (Female)	Male – 1<br>Female – 2
Race (race1v)	1 (white), 2 (black) 3-97 (specific races), 98 (other), 99 (unknown)	White – 1<br>Black – 2<br>Other – 3-98
Detection Stage (hst_stga (prior to 2016) & dsrpsg (2016))	hst_stga description:<br>0 (In situ —A noninvasive neoplasm; a tumor which has not penetrated the basement membrane nor extended beyond the epithelial tissue)<br>1 (Localized —An invasive neoplasm confined entirely to the organ of origin. It may include intraluminal extension where specified)<br>2 (Regional —A neoplasm that has extended 1) beyond the limits of the organ of origin directly into surrounding organs or tissues; 2) into regional lymph nodes by way of the lymphatic system; or 3) by a combination of extension and regional lymph nodes)<br>4 (Distant —A neoplasm that has spread to parts of the body remote from the primary tumor either by direct extension or by discontinuous metastasis to distant organs, issues, or via the lymphatic system to distant lymph nodes)<br>8 (Localized/Regional – Only used for Prostate cases)<br>9 (Unstaged —Information is not sufficient to assign a stage)	In situ – 0 (hst_stga) & 0x (dsrpsg)<br>Localized – 1 (hst_stga) & 1x (dsrpsg)<br>Regional – 2 (hst_stga) & 2x/3x (dsrpsg)<br>Distant – 4 (hst_stga) & 4x (dsrpsg)<br>Unknown – 9 (hst_stga) & 99/88/OC (dsrpsg)<br><br>For prostate:<br>Localized/Regional – 8 (hst_stga) & 1x/2x/3x (dsrpsg)
Grade Differentiation (grade)	1 (Grade I; grade i; grade 1; well differentiated; differentiated, NOS), 2 (Grade II; grade ii; grade 2; moderately differentiated; moderately differentiated; intermediate differentiation), 3 (Grade III; grade iii; grade 3; poorly differentiated; differentiated), 4 (Grade IV; grade iv; grade 4; undifferentiated; anaplastic), 5 (T-cell; T-precursor), 6 (B-cell; Pre-B; B-Precursor), 7 (Null cell; Non T-non B), 8 (NK cell (natural killer cell)), 9 (cell type not determined, not stated or not applicable)	Well – 1<br>Moderate – 2<br>Poor – 3<br>Undifferentiated – 4<br>Unknown – 9
Surgery (ss_surg (for cases prior to 1998) & surgprif (for cases from 1998-2016))	00 (None, no surgical procedure of primary site, diagnosed at autopsy only), 10-98 (site specific codes), 99 (unknown if surgery performed; death certificate only)	Yes – 01-98<br>No – 00
Radiotherapy (radiatnr)	0 (None/Unknown, diagnosed at autopsy), 1 (Beam radiation), 2 (Radioactive implants), 3 (Radioisotopes), 4 (Combination of 1 with 2 or 3), 5 (Radiation, NOS – method or source not specified), 6 (Other radiation – 1973-1987 cases only), 7 (Patient or patient's guardian refused radiation	Yes – 1,2,4,3,4,5,6<br>No – 0,7,8

	therapy), 8 (Radiation recommended, unknown if administered)	
Chemotherapy<br><br>(chemo_rx_rec)	0 (None/Unknown), 1 (Yes)	Yes – 1<br>No – 0

Table S6. Breakdown of mucinous cases in SEER (1975-2016), both analyzed and not analyzed.

Mucinous Cases in SEER		(1975-2016)		(1992-2016)	
**Site**	**SEER Site Recode (siterwho) ICD-O-3/WHO 2008**	**Count**	**% Total**	**Count**	**% Total**
**Sites Analyzed**		**161,471**	**95.21**	**130,515**	**95.65**
Colon	21041 (Cecum), 21043, (Ascending Colon), 21044 (Hepatic Flexure), 21045 (Transverse Colon), 21046 (Splenic Flexure), 21047 (Descending Colon), 21048 (Sigmoid Colon), 21049 (Large Intestine, NOS)	65,747	38.77	52,275	38.31
Breast	26000 (Breast)	31,502	18.57	26,835	19.67
Lung	22030 (Lung and Bronchus)	15,744	9.28	12,674	9.29
Rectum	21051 (Rectosigmoid Junction), 21052 (Rectum)	14,355	8.46	11,110	8.14
Pancreas	21100 (Pancreas)	8629	5.09	6922	5.07
Ovary	27040 (Ovary)	5555	3.28	4591	3.36
Stomach	21020 (Stomach)	4854	2.86	3169	2.32
Appendix	21042 (Appendix)	4126	2.43	3838	2.81
Uterus	27020 (Corpus Uteri), 27030 (Uterus, NOS)	2735	1.61	2278	1.67
Gallbladder/Biliary	21080 (Gallbladder), 21090 (Other Biliary)	1963	1.16	1524	1.12
Cervix	27010 (Cervix Uteri)	1360	0.80	1198	0.88
Small Bowel	21030 (Small Intestine)	1196	0.71	1002	0.73
Esophagus	21010 (Esophagus)	1181	0.70	1050	0.77
Prostate	28010 (Prostate)	1044	0.62	856	0.63
Urinary Bladder	29010 (Urinary Bladder)	829	0.49	644	0.47
Anus	21060 (Anus, Anal Canal and Anorectum)	651	0.38	549	0.40
**Sites Not Analyzed**		**8124**	**4.79**	**5931**	**4.35**
Oral Cavity/ Pharynx	20010 (Lip), 20020 (Tongue), 20030 (Salivary Gland), 20040 (Floor of Mouth), 20050 (Gum and Other Mouth), 20060 (Nasopharynx), 20070 (Tonsil), 20090 (Hypopharynx), 20100 (Other Oral Cavity and Pharynx)	208	0.11	132	0.08
Liver	21071 (Liver), 21072 (Intrahepatic Bile Duct)	220	0.13	169	0.13
Peritoneum	21110 (Retroperitoneum), 21120 (Peritoneum, Omentum and Mesentery)	195	0.11	183	0.14
Other Digestive	21130 (Other Digestive Organs)	778	0.46	653	0.48
Respiratory System	22010 (Nose, Nasal Cavity and Middle Ear), 22020 (Larynx), 22060 (Trachea, Mediastinum and Other Respiratory Organs)	100	0.07	60	0.03
Soft Tissue	24000 (Soft Tissue Including Heart)	14	0.01	14	0.01
Skin	25020 (Other Non-Epithelial Skin)	567	0.33	517	0.38
Female Genital System	27050 (Vagina), 27060 (Vulva), 27070 (Other Female Genital Organs)	216	0.13	173	0.13
Male Genital System	28020 (Testis), 28030 (Penis), 28040 (Other Male Genital Organs)	8	0.01	8	0.01
Urinary System	29020 (Kidney and Renal Pelvis), 29030 (Ureter), 29040 (Other Urinary Groups)	213	0.12	177	0.13
Eye and Orbit	30000 (Eye and Orbit)	9	0.01	9	0.01
Endocrine System	32010 (Thyroid), 32020 (Other Endocrine including Thymus)	28	0.02	22	0.02
Miscellaneous	37000 (Miscellaneous)	5568	3.28	3814	2.80

© 2020 by the authors. Submitted for possible open access publication under the terms and conditions of the Creative Commons Attribution (CC BY) license (http://creativecommons.org/licenses/by/4.0/).
